# High-Throughput Mass Spectrometric Analysis of the Whole Proteome and Secretome From *Sinorhizobium fredii* Strains CCBAU25509 and CCBAU45436

**DOI:** 10.3389/fmicb.2019.02569

**Published:** 2019-11-12

**Authors:** Hafiz Mamoon Rehman, Wai-Lun Cheung, Kwong-Sen Wong, Min Xie, Ching-Yee Luk, Fuk-Ling Wong, Man-Wah Li, Sau-Na Tsai, Wing-Ting To, Lok-Yi Chan, Hon-Ming Lam

**Affiliations:** Center for Soybean Research of the State Key Laboratory of Agrobiotechnology and School of Life Sciences, The Chinese University of Hong Kong, Shatin, Hong Kong

**Keywords:** *Sinorhizobium fredii*, proteome, secretome, nodulation outer proteins, soybean

## Abstract

*Sinorhizobium fredii* is a dominant rhizobium on alkaline-saline land that can induce nitrogen-fixing symbiotic root nodules in soybean. Two *S. fredii* strains, CCBAU25509 and CCBAU45436, were used in this study to facilitate in-depth analyses of this species and its interactions with soybean. We have previously completed the full assembly of the genomes and detailed transcriptomic analyses for these two *S. fredii* strains, CCBAU25509 and CCBAU45436, that exhibit differential compatibility toward some soybean hosts. In this work, we performed high-throughput Orbitrap analyses of the whole proteomes and secretomes of CCBAU25509 and CCBAU45436 at different growth stages. Our proteomic data cover coding sequences in the chromosome, chromid, symbiotic plasmid, and other accessory plasmids. In general, we found higher levels of protein expression by genes in the chromosomal genome, whereas proteins encoded by the symbiotic plasmid were differentially accumulated in bacteroids. We identified secreted proteins from the extracellular medium, including seven and eight Nodulation Outer Proteins (Nops) encoded by the symbiotic plasmid of CCBAU25509 and CCBAU45436, respectively. Differential host restriction of CCBAU25509 and CCBAU45436 is regulated by the allelic type of the soybean Rj2(Rfg1) protein. Using sequencing data from this work and available in public databases, our analysis confirmed that the soybean Rj2(Rfg1) protein has three major allelic types (Rj2/rfg1, rj2/Rfg1, rj2/rfg1) that determine the host restriction of some *Bradyrhizobium diazoefficiens* and *S. fredii* strains. A mutant defective in the type 3 protein secretion system (T3SS) in CCBAU25509 allowed this strain to nodulate otherwise-incompatible soybeans carrying the rj2/Rfg1 allelic type, probably by disrupting Nops secretion. The allelic forms of NopP and NopI in *S. fredii* might be associated with the restriction imposed by Rfg1. By swapping the NopP between CCBAU25509 and CCBAU45436, we found that only the strains carrying NopP from CCBAU45436 could nodulate soybeans carrying the rj2/Rfg1 allelic type. However, no direct interaction between either forms of NopP and Rfg1 could be observed.

## Introduction

Rhizobia are Gram-negative bacteria that form symbiotic root nodules in leguminous plants. They reduce atmospheric dinitrogen to ammonia which can then be efficiently used by host plants for growth and development, in return for carbon and other nutrients provided by their hosts. Thus, they constitute an important mechanism for sustainable agriculture by reducing the dependence on synthetic nitrogen fertilizers (Dixon and Kahn, 2004).

Most of the recognized legume-nodulating rhizobia belong to the genera *Rhizobium*, *Mesorhizobium*, *Sinorhizobium* (*Ensifer*), and *Bradyrhizobium* within the α-proteobacteria class (Sugawara et al., 2013). The genus *Sinorhizobium* can induce nodule formation on the roots of many host plants from the *Leguminosae* family. *Sinorhizobium meliloti*, and its close relative, *Sinorhizobium medicae*, can establish symbiosis with *Medicago truncatula* and *Medicago sativa*, while *Sinorhizobium saheli* and *Sinorhizobium terangae* form root and stem nodules with *Sesbania* or *Acacia* (woody legumes). *Sinorhizobium fredii*, on the other hand, has a very wide range of hosts, representing all three subfamilies of the *Leguminosae* family, including cultivated (*Glycine max*) and wild (*G. soja*) soybeans (Sugawara et al., 2013).

*Bradyrhizobium* (species *japonicum* and *diazoefficiens*) and *Sinorhizobium* (species *fredii*) are the two major genera that can form nodules with the important crop soybean. Soybean-nodulating *B. japonicum* and *B. diazoefficiens* are more prominent in acidic soils and exhibit high nitrogen fixation capacity (Man et al., 2008; Tian et al., 2012), and they have long been used as inoculants in the field to improve yield and reduce fertilizer use (Paau, 1989). On the other hand, *S. fredii* are often found in alkaline soils in Asia (Han et al., 2009) and could perform efficient nitrogen fixation with some Chinese soybean cultivars (Muñoz et al., 2016). Yet, the use of *Sinorhizobium* as inoculant is not very widespread in Asia.

High-quality reference genomes of *S. fredii* with good annotation have been released in recent years (Schmeisser et al., 2009; Schuldes et al., 2012; Weidner et al., 2012; Jiao et al., 2018; Dang et al., 2019). While the transcriptome of prokaryote RNA is polycistronic in nature, prediction of coding sequences (CDSs) by open reading frames (ORFs) in operons, RNA sequencing, and sequence homology searches are only indicative. Furthermore, since protein abundance and varieties are dependent on the rate of mRNA translation as well as protein degradation and post-translational modifications, transcriptomic analyses can only partially explain the proteome (Muers, 2011). High-throughput proteomics has therefore provided evidence to support the existence of potential coding sequences and provided quantitative measurement of proteins and their variants to better correlate with the biochemical functions under different conditions. Over the last 20 years, proteomics based on mass spectrometry (MS) has contributed significantly to protein studies by facilitating protein identification and quantification. MS supports both relative and absolute protein measurement at a larger scale without the need to generate antibodies (Liu et al., 2016).

In addition to the free-living forms, rhizobia also exist as bacteroids in the host cells inside root nodules. This unique cell stage undergoes massive changes in its transcriptome and proteome under the influence of the host plant. Study of the bacteroid proteome which is encapsulated by the host cell could be challenging (Marx et al., 2016), as it will require either the analysis of complex host-symbiont co-proteome or the isolation of bacteroids in order to study the bacteroid-specific proteome.

Furthermore, the initiation of the nodulation process requires signal exchange between the host and the bacteria, which involves nodulation factors (lipo-chitooligosaccharides), surface carbohydrates and extracellular proteins (Krishnan et al., 2003; Okazaki et al., 2013; Staehelin and Krishnan, 2015; Sugawara et al., 2018). Previous studies characterizing secreted proteins relied largely on forward genetic studies (Sugawara et al., 2018) and gel-based proteomics (Rodrigues et al., 2007) which limited the throughput and scope of the studies. Eight nodulation outer proteins (Nops) (NopA, NopB, NopC, NopD, NopI, NopP, NopL, and NopX) have been detected in *S. fredii* strain HH103 in the extracellular medium based on precipitated proteins in response to genistein induction (Rodrigues et al., 2007). In more recent years, several Nops have been functionally characterized in *S. fredii* for their involvement in nodulation processes and the findings were reviewed by Staehelin and Krishnan (2015). Thus far, little is known about the biochemical functions of the secretomes of rhizobia. Hence, an extensive catalog of secreted proteins and the effective comparison of secretomes between rhizobium strains would greatly help us understanding the roles of these proteins during host-rhizobium interactions.

Host-specific nodulation may rely on Nops, i.e., rhizobial effectors, translocated into the host cytoplasm through the type 3 protein secretion system (T3SS) (Krishnan and Pueppke, 1993). The T3SS is common to many bacterial pathogens targeting animals and plants (Staehelin and Krishnan, 2015). T3SS effector proteins have been well-characterized in various *S. fredii* strains, NGR234, USDA257, and HH103 (Krishnan et al., 2003; Marie et al., 2004; López-Baena et al., 2016). The effectors secreted by T3SS, upon entering the plant cell, may lead to effector-triggered immunity (ETI) mediated by resistance proteins (R proteins) in plants (Hurley et al., 2014). Rhizobial Nops can also help determine host specificity, such as in the cases of *Sinorhizobium*- and *Bradyrhizobium*-soybean interactions (Yang et al., 2010; Sugawara et al., 2018). Earlier, it was reported that allelic types of the soybean R protein Rj2(Rfg1) protein could determine host restriction of *B. diazoefficiens* and *S. fredii* (Yang et al., 2010). For example, soybean carrying the dominant type of the *Rfg1* gene can restrict the nodulation by *S. fredii* USDA193 (Fan et al., 2017).

In response to flavonoids derived from host plants, various transcription factors (e.g., NodD1, NodD2, and *ttsI*) can trigger activation of genes (e.g., *rhc*, *nol*, and *nop*) responsible for T3SS (Wassem et al., 2008). In *S. fredii*, approximately 15 genes have been predicted to encode for the T3SS Nops (Kimbrel et al., 2013).

The mutants *rhcJ* and *ttsI* of *B. japonicum* USDA122 fail to secrete Nops and were able to nodulate otherwise non-compatible soybean plants, suggesting that effectors secreted via T3SS trigger incompatibility with soybean hosts (Tsukui et al., 2013). The determining factor in *B. diazoefficiens* strain USDA122 has been shown to be the NopP effector protein. Mutations in the *nopP* gene are tightly associated with host compatibility in soybean expressing the Rj2 R protein (Sugawara et al., 2018).

A negative effect on the early stages of soybean nodulation was observed upon the inactivation of the *S. fredii* HH103 T3SS, which suggests that T3SS is required to suppress plant-generated immune responses for successful nodulation in some cases (Jiménez-Guerrero et al., 2015). Random Tn5 insertion mutations in the T3SS gene cluster in an incompatible strain of *S. fredii* (CCBAU25509) led to nodule formation in *G. max* C08, suggesting a significant role of insertion sequences in the adaptive evolution of rhizobial compatibility (Zhao et al., 2017). However, mutations in a number of nop genes have been found affecting symbiosis (Staehelin and Krishnan, 2015; López-Baena et al., 2016). Recently, NopI of *S. fredii* HH103 was also found to determine nodulation numbers in soybean (Jiménez-Guerrero et al., 2017). In *S. fredii* HH103, NopI, and NopP show 48% amino acid sequence identity (Jiménez-Guerrero et al., 2017).

Here we make use of the high-throughput Orbitrap system to study the proteomes of two *S. fredii* strains, CCBAU45436 and CCBAU25509, during mid-log, stationary phase and in bacteroids using different soybean hosts. We have previously completed their genome assembly and transcriptome analyses (Jiao et al., 2018) and the data could serve as the basis for the proteomic analyses. The purpose is to build two important reference proteomes for these two strains of *S. fredii* that have been reported to exhibit differential host specificities toward soybean expressing the R protein, Rfg1 (Qi et al., 2014; Muñoz et al., 2016; Zhao et al., 2017). In addition, we also investigated the secretomes of both strains to get insights into the Nops that may be associated with host compatibility. The methods previously reported for Nops detection in the extracellular medium required specific antibodies that are labor-intensive and time-consuming to produce while our adapted method for detecting effector proteins is faster and more reliable, all without the need for generating antibodies. A randomly generated Tn5-inserted *rhcN* mutant mitigated the host incompatibility with *G. max* C08 by suppressing the secretion of T3SS-related Nops and disrupting the expressions of pilus assembly-associated genes. Furthermore, we have also identified NopP as a key component in determining genotype-specific nodulation of soybean.

## Materials and Methods

### Cultivation of Bacteria and Plants

*Sinorhizobium fredii* CCBAU25509 and CCBAU45436 were cultured at 28°C in yeast mannitol agar (YMA) medium with nalidixic acid (25 mg/L). Both wild type CCBAU25509 and CCAU45436 are resistant to nalidixic acid. Plant growth and inoculation was also carried out in accordance with the method described previously (Jiao et al., 2018). The surface-sterilized seeds of *G. max* (accession C08) and *Glycine soja* (accession W05) were germinated in vermiculite wetted with autoclaved water in the greenhouse. Following germination, 1 ml each of CCBAU25509 or CCBAU45436 suspension culture (OD_600_ = 0.8) per plant was inoculated onto the seedlings. Bacteroids were collected in 15 ml of extraction buffer [10 mM DTT, 300 mM sucrose, 10 mM phosphate buffer pH 7.0, 2 mM MgCl_2_ and 2% (w/v) PVP-40] by extensive grinding of 2–5 g nodules in a pre-cooled mortar and pestle and later purified on a Percoll gradient according to the method described previously (Li et al., 2013). The bacteroids were collected from the nodules, then washed with Milli-Q water and stored at −80°C until use.

### Extraction of Total and Secreted Proteins

For the extraction of total proteins, *S. fredii* strains were grown in the YMA medium at 28°C until mid-log (∼15 h culture, OD_600_ = 0.7) and stationary phase (∼48 h culture, OD_600_ = 1.4), and the bacteria were collected by centrifugation at 4000 × *g*. The pellets obtained were washed twice in phosphate buffered saline (PBS) (pH 7.2) and subjected to lysis in a buffer composed of 2% SDS in 20 mM Tris-HCl (pH 8.8) at 95°C at 500 rpm in a Thermomixer for 30 min (Eppendorf, Germany) (Tanca et al., 2013). The protein-containing supernatant was extracted for 5 min at 12000 × *g* by centrifugation. Proteins were precipitated using chilled acetone at −20°C for 4 h and the resulting pellet was dissolved in 50 mM Tris-HCl buffer (pH 8.5) containing 8M urea. The same protocol was adopted for the extraction of proteins from bacteroids from C08 and W05 nodules. In total, three replicates were used to perform whole proteome analysis.

Extracellular proteins were induced from bacterial strains grown in 50 ml of RMS medium in a 500 ml flask (Broughton et al., 1986) with a final concentration of 10 μM genistein on an orbital shaker (180 rpm) for 28 h (about 10^9^ bacteria ml^–1^) at 28°C (Jiménez-Guerrero et al., 2017). Two successive centrifugation steps at 4°C for 4000 × *g* and 8000 × *g* for 30 min were used to remove bacterial cells. The supernatants were mixed with 3 volumes of chilled acetone and kept for 24 h at −20°C. The mixtures were centrifuged at 22,000 × *g* at 4°C for 45 min and the resulting pellets were dried and resuspended in 300 μl of 8M urea in Tris-HCl buffer (pH 8). The same volume of extracted extracellular proteins were separated on NuPAGE^TM^ 4–12% bis-tris protein gels with MES running buffer. SilverXpress^TM^ silver staining kit was used for visualizing the separated proteins (Thermo Scientific, United States). For secretome analysis, the experiment was repeated two times.

Digestion of proteins was performed using SMART digest^TM^ trypsin kit (Thermo Scientific, United States) in solution. The reduction and alkylation of the tryptic peptides was achieved with the use of 10 mM dithiothreitol (DTT) at 56°C for 30 min and 25 mM iodoacetamide at room temperature for 25 min. The digested peptides were purified using Pierce^TM^ C-18 spin columns and finally dissolved in 0.1% formic acid (FA).

### Orbitrap-Based LC-MS/MS Analysis

MS analysis was performed using an Orbitrap Fusion^TM^ Lumos^TM^ Tribrid^TM^ Mass Spectrometer (Thermo Scientific, United States) interfaced with an LC UltiMate 3000 RSLCnano system. The peptide separation was carried out at 50°C with C-18 μ-precolumn (300−μm i.d. × 5 mm) followed by Acclaim^TM^ PepMap^TM^ RSLC, nanoViper C-18 column, 75 μm × 25 cm (Thermo Scientific, United States) at a flow rate of 0.3 μl/min, using mobile phase A [98% H_2_O, 1.9% acetonitrile (ACN) with 0.1% FA] and mobile phase B (98% ACN, 1.9% H_2_O with 0.1% FA). The following LC gradient was used to detect secretome proteins: 100% A for the initial 5 min, then 0% to 6% B for 3 min, then 6% B to 30% B for 42 min, then 30% B to 45% B for 10 min, then 45% B to 60% B for 10 min, then 60% B to 80% B for 5 min and an additional 5 min at 80% B followed by final re-equilibrium with 100% A for 10 min. We used the following LC gradient profile (100% A for the initial 5 min, then 0% B to 6% B for 3 min, then 6% B to 18% B for 40 min, then 18% B to 30% B for 10 min, then 30% B to 80% B for 2 min, then 80% B for an additional 5 min followed by re-equilibrium with 100% A for 10 min) for total protein detection.

The Orbitrap was set up in a data-dependent MS/MS mode under direct control of the Xcalibur software (version 4.1), where a full-scan spectrum (from 375 to 1500 *m*/*z*) was followed by tandem mass spectra (MS/MS). The instrument was operated in positive mode with a spray voltage of 2 kV, a capillary temperature of 300°C, and was calibrated before measurements. Full scans were performed in the Orbitrap with a resolution of 60,000 at 400 *m*/*z*, with a precursor ion selection (AGC > 4.0e5) and ion charge > 1. Higher energy collisional dissociation (HCD), performed at the far side of the C−trap, was chosen as the fragmentation method, by applying a 30% value for normalized collision energy, an isolation window of *m*/*z* 1.6, with a maximum injection time of 250 milli seconds (ms) and Orbitrap resolution of 15,000.

The Proteome Discoverer platform (version 2.3; Thermo Scientific, Germany), interfaced with an in−house Sequest server, was used for data parsing and protein identification, according to the following criteria: in-house database of CCBAU25509 and CCBAU45436, Enzyme Trypsin, Maximum Missed Cleavage Sites 2, Precursor Mass Tolerance 10 ppm, Fragment Mass Tolerance 0.2 Da, Cysteine Carbamidomethylation as Static modification, N−terminal Acetylation and oxidation as dynamic modifications. The Percolator algorithm was used for peptide validation [peptide confidence: *q*−value < 0.01, corresponding to false discovery rate (FDR) < 0.01], and only rank 1 peptides were considered. Peptide and protein grouping according to Proteome Discoverer’s algorithms were allowed, applying strict maximum parsimony principle. A Label-free quantification (LFQ) analysis was performed with three replicates for each treatment for whole proteome analysis.

### Phylogenomic Analysis

In this study, 16S rRNA sequences were retrieved from 56 rhizobial genomes (Supplementary Table S1), including 10 *S*. *fredii* strains, 10 *S*. *meliloti* strains, 6 *S. medicae* strains, 9 *B. japonicum* strains, 5 *Mesorhizobium* sp. strains, 6 *B. diazoefficiens* strains, 4 *B. elkanii* strains, and 6 *R. leguminosarum* strains using the EzBioCloud database^1^ (Yoon et al., 2017). The evolutionary history was inferred by using the Maximum Likelihood method based on the Tamura-Nei model (Tamura and Nei, 1993) in MEGA7 (Kumar et al., 2016).

### Genome- and Transcriptome-Wide Identification of Nops in Selected Genomes

For deeper analyses of the *nop* genes in CCBAU25509 and CCBAU45436, we retrieved the predicted *nop* sequences from *S. fredii* NGR234, USDA257, and USDA207 (Kimbrel et al., 2013) for use in our search. To predict the upstream *tts* box sequences reported for *nop* gene regulation, we constructed HMMs (Hidden Markov Models) for *tts* box sequence homologs in CCBAU25509 and CCBAU45436 accordingly (Marie et al., 2004; Songwattana et al., 2017). Nops detected in the extracellular medium from *S. fredii* CCBAU25509 and CCBAU45436 were further analyzed for the presence or absence of the eight identified Nops in the 56 selected genomes using the BLAST tool available in the EzBioCloud database with a cutoff of e-value ≤ 1e^–10^ and protein sequence identity ≥ 40%. In complement of this, the proteins were search against NCBI database using BLASTP with the same cutoff. A heatmap was generated based on presence of a *nop* gene sequence in all 56 selected genomes. The protein sequences obtained were screened and aligned using the MEGA7^2^ MUSCLE alignment tool. The information associated with the detected Nops, such as the CDS, the name of the rhizobial strain and genomic location, was also recorded. To check the growth stage-specific expression of all eight Nops during the mid-log and stationary phases and in the collected bacteroids, the RNA-Seq dataset was further analyzed using Fragments Per Kilobase of transcript, per Million mapped reads (FPKM) values collected from transcriptome datasets available (Jiao et al., 2018). The expression of the *nopP* and *nopI* genes was further confirmed by qPCR using One Step SYBR Prime Script^TM^ RT-PCR Kit II (Takara Bio, Inc., Japan), according to the manufacturer’s instruction, and primers are listed in Supplementary Table S2.

### Genotyping and Phenotyping of Soybean Accessions

Full length or a partial fragment of the *Rj2*(*Rfg1*) gene of 22 soybean accessions (Lam et al., 2010; Muñoz et al., 2016) were amplified from genomic DNA using iProof polymerase (BioRad) according to the manufacturer’s instruction. Primers for the amplification and sequencing are listed in Supplementary Table S2. For the nodulation test, surface-sterilized seeds were sown on wetted vermiculite followed by the addition of 1 ml overnight culture of CCBAU25509 or USDA122 per seed. Formation of effective nodules were confirmed 28 days post-inoculation. Re-sequencing data were retrieved from a previous publication (Zhou et al., 2015). Only the single-nucleotide polymorphisms (SNPs) at the seven previously reported polymorphic sites (E452K, I490R, Q731E, E736N, P743S, E756D, and R758S) distinguishing different alleles of Rj2(Rfg1) were analyzed (Yang et al., 2010). SNP on the 758^th^ amino acid residue was missing in the dataset and was not included in the analyses. Accessions with missing data or heterozygous SNPs at the polymorphic sites of interest were discarded. Allelic forms were determined according to Yang et al. (2010).

### Generation, Screening, and Plant Assays of the *rhcN:Tn5* Mutant

Bi-parental mating was performed between the donor *E. coli* S17-1 λpir (Biomedal Life Science, Spain) harboring pUTmini-Tn5 km (Biomedal Life Science, Spain) and the recipient *S. fredii* CCBAU25509 by mixing their cultures in a ratio of 1:3 (Tsurumaru et al., 2008) on TY agar for conjugation and mutagenesis. Mutants were selected on a TY agar plate supplemented with nalidixic acid (50 mg/L) and kanamycin (50 mg/L). A total number of > 1.27 × 10^6^ individual clones were inoculated into 2-day-old C08 seedlings. Nodules were collected 28 days after inoculation. The collected nodules were surface-sterilized with 5% household bleach and washed thoroughly with autoclaved Milli-Q water. Mutants were isolated aseptically by inserting autoclaved toothpicks into the core of the nodules and streaking on TY agar with nalidixic acid (50 mg/L) and kanamycin (50 mg/L) for selection.

The isolates grown on the selection medium were further subjected to PCR screening with Tn5 transposon-specific primers and one pair of CCBAU25509-specific primers (Supplementary Table S2) to eliminate possible contamination from other antibiotic-resistant strains. The PCR-confirmed mutants were re-inoculated into C08 to allow nodulation. Genomic DNA (gDNA) of the confirmed mutants were extracted using the CTAB buffer protocol. The Tn5 insertion site was determined by Y-linker PCR. In brief, gDNA was digested with *Nla*III restriction endonuclease. Forty nanograms of the resulting gDNA were used to ligate with 1 μg of Y-linker in a 20 μl reaction at 16°C overnight (Kwon and Ricke, 2000). The ligated product was then amplified by PCR with Y-linker- and transposon-specific primers (Supplementary Table S2) for the selective amplification of the 5′-flanking region of the transposon and cloned into pMD20 T-vector (Takara Bio, Inc., Japan) and the successful cloning was confirmed by sequencing. The chlorophyll contents of soybean plants inoculated with wild type and mutant CCBAU25509 were measured in a portion of the leaf excised from the first trifoliate and calculated based on the recorded fresh weight, following a method described previously (Millard and Robinson, 1987).

### Gene Expression Analysis

Wild type CCBAU25509 and the obtained *rhcN:Tn5* mutant were grown in 50 ml TY medium and induced with 20 μM genistein or an equivalent volume of DMSO as control. Cultures were collected at OD_600_ = 0.4 and pelleted by centrifugation at 5000 × *g*. The RNA was extracted using TRIzol reagent (Invitrogen, United States) according to the manufacturer’s protocol with a modification in the lysis step in which ∼100 μl of glass powder was added per ml of TRIzol reagent. DNase I-treated RNA was diluted 10-fold for qPCR using One Step SYBR^®^ Prime Script^TM^ RT-PCR Kit II (Takara Bio, Inc., Japan) according to the manufacturer’s instruction, except that annealing and extension was performed at 55.7°C. Primer sequences for RT-qPCR can be found in Supplementary Table S2. The amplification efficiencies and specificities of the primers used for qPCR were validated with primer efficiency assays and melt curve analyses. *GyrA* was used as the housekeeping gene for normalization.

### Generation of *nopP*-Exchanged Mutants of CCBAU25509 and CCBAU45436

The *nopP* coding sequences with ∼1 kb upstream/downstream were amplified from the genomic DNA of CCBAU25509 and CCBAU45436 and cloned between the *Xba*I and *Pst*I sites of pK18mobsacB (ATCC^®^ 87097^TM^). Swapping of the *nopP* genes was done using an established protocol (Schafer et al., 1994) with slight modifications. In brief, an 8,714 bp construct containing the 3,013 bp *nopP* fragment from CCBAU25509 (*nopP*_25509_) was transferred into CCBAU45436, and vice versa, by bi-parental mating using *E*. *coli* S17-1 λpir. TY agar supplemented with nalidixic acid (50 mg/L) and kanamycin (50 mg/L) was used for the selection of the first crossing-over event while TY agar supplemented with nalidixic acid (50 mg/L) and 10% sucrose was used to select for the second crossing over. Mutants with successfully exchanged *nopPs* were confirmed by sequencing. The resulting CCBAU25509*nopP*_45436_ and CCBAU45436*nopP*_25509_ were used to inoculate C08 for the assessment of nodulation phenotype. The identities of the bacteria within the nodules were confirmed using PCR to rule out possible cross-contamination. Numerical data were analyzed using one-way ANOVA followed by Tukey’s *post hoc* test using GraphPad Prism (ver. 8.1.1). Primers for generating and screening of the mutants can be found in Supplementary Table S2.

### Yeast Two-Hybrid Assay

Full-length and selected domains of *Rfg1* cDNA were amplified from C08 and W05 and were subcloned into pGBKT7 (Clontech, United States). *nopP* coding sequences were amplified from both CCBAU25509 and CCBAU45436 and were cloned into pGADT7-rec (Clontech, United States). Primers for the amplification of target sequences can be found in Supplementary Table S2. The pGBKT7 and pGADT7-rec constructs were transformed into Y2H gold and Y187 (Takara Bio, Inc., Japan), respectively, using lithium acetate/polyethylene glycol method (Gietz and Schiestl, 2007). Yeast mating and interaction analyses were done according to Yeast Protocols Handbook (Clontech, United States).

## Results

### Orbitrap-Based Total Proteomics of CCBAU25509 and CCBAU45436

We performed a comprehensive proteome profiling of CCBAU45436 and CCBAU25509 at mid-log and stationary phases, and in bacteroids with three biological replicates for each condition. The peptides obtained from the tryptic digestion of extracted proteomes were analyzed by Orbitrap Fusion^TM^ Lumos^TM^ Tribrid^TM^ Mass Spectrometer coupled to a nano-liquid chromatography (LC).

**FIGURE 1:**
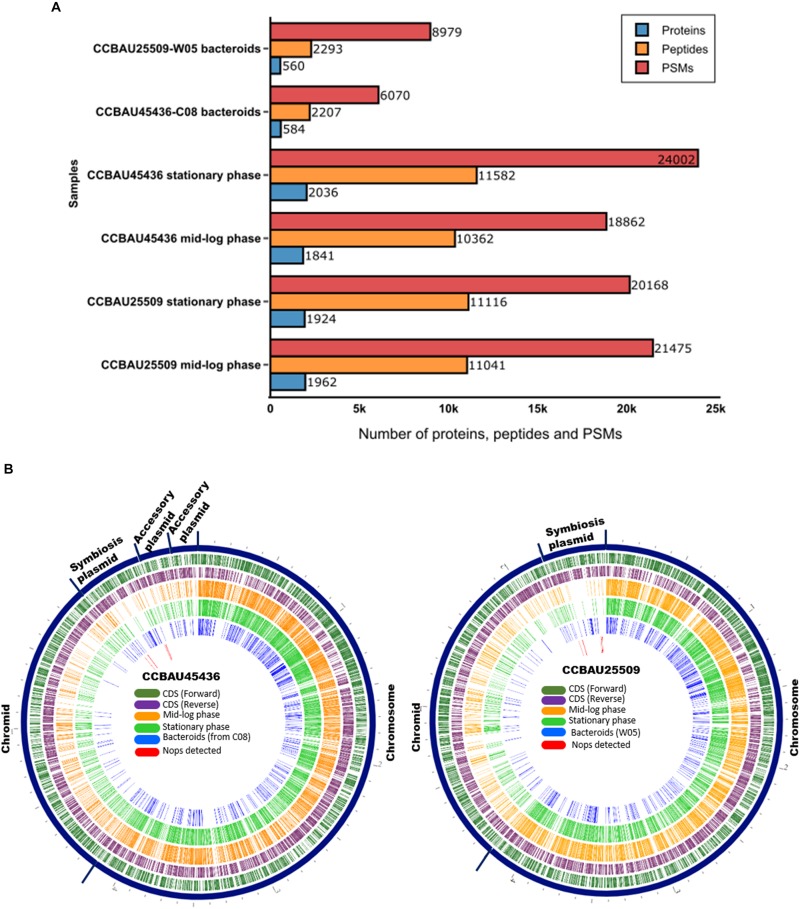
Proteomes of *Sinorhizobium fredii* CCBAU25509 and CCBAU45436. **(A)** Number of identified proteins, peptides, and peptide spectrum matches (PSMs) from CCBAU25509 and CCBAU45436 at various growth stages in one of the three replicates. Bacteroids of CCBAU25509 and CCBAU45436 were obtained by inoculating these strains into their compatible soybean hosts, W05 and C08, respectively. **(B)** Circular views of detected proteins overlaid on the genomic locations of the encoding genes.

On average, during the mid-log and stationary phases, we detected nearly 20,000 peptide spectrum matches (PSMs) corresponding to 11,000 peptides, or 2,000 proteins in each replicate with the false discovery rate (FDR) of *q* < 0.01 (Fisher’s exact test) (Figure 1A and Supplementary Tables S3, S4). Almost 600 proteins were detected from bacteroids collected from the nodules of *G. max* (C08) and *G. soja* (W05) plants inoculated with *S. fredii* CCBAU45436 and CCBAU25509, respectively, after 28 days (Figure 1A and Supplementary Tables S3, S4). Nodules could not be formed on C08 roots inoculated with CCBAU25509 due to the presence of the *Rfg1* allele in C08, which rendered C08 incompatible with the rhizobium. The constructed circular proteomic and genomic maps of both strains provide a bird’s-eye view of the translating core and accessory genomes of *S. fredii* at various growth stages (Figure 1B).

During all three growth stages, a large proportion of the detected proteomes were encoded by the chromosome-borne CDSs of each strain (Figure 1B). This is consistent with the observation of a previous proteomic study of *S. medicae* strain (Berrabah et al., 2018). All proteome datasets had proteins spanning a range of molecular weights and isoelectric points (Supplementary Tables S3, S4). ABC transporter proteins, tricarboxylic acid (TCA) cycle proteins, cold shock proteins, DUF (domain of unknown function) proteins, ferredoxin family proteins, heat shock proteins, nitrogen fixation and regulatory proteins, phosphate transport proteins, outer membrane lipoproteins, and transcriptional regulator proteins made up the key families of proteins detected in the bacteroids collected from C08 and W05 nodules. The phasin family and the chaperonin proteins were abundantly found in all samples analyzed based on the number of peptides.

Gene ontology (GO) annotation analyses of all the identified proteins showed that the major portion of the proteome was found in the cytoplasm, integral component of membrane, periplasmic space, ribosome, plasma membrane, and cell membrane components of the cell at all growth stages (Supplementary Figure S1). At the molecular level, most of the proteome was involved in ATP binding, metal ion binding and transferase activity, oxidoreductase activity, as the structural constituent of ribosome, DNA-binding transcription factor activity, ATPase activity, hydrolase activity, rRNA binding, and pyridoxal phosphate binding, in both rhizobial strains at all three growth stages. A significant portion of the proteome was also involved in metabolic activities at all three growth stages, such as the regulation of transcription, carbohydrate metabolic processes, phosphorelay signal transduction system, transmembrane transport, cell redox homeostasis, TCA cycle, and nitrogen compound metabolic processes. On the other hand, a major portion of the proteins from the bacteroids were found to be involved in nitrogen fixation when classified according to biological processes.

The reproducibility among replicates was based on the calculated Log_10_ abundance of peptides using the available LFQ consensus and processing workflow in the Proteome Discoverer software (version 2.3). The LFQ peptides between each replicate had almost the same mean value, showing a high degree of reproducibility among the samples (Supplementary Figure S2). The abundantly expressed stage-specific peptides based on *p*-value < 0.05 are found in a comparison between mid-log and stationary phase LFQ datasets (Supplementary Figure S2).

By comparing the flagellar export proteins in the mid-log phase versus the stationary phase, carbonic anhydrase, DUF971 domain-containing protein, nitrogen regulatory protein P-II and protein-export chaperone, SecB, chaperonin, and the phasin family of proteins were once again found to be the most highly expressed proteins (Supplementary Figure S3 and Table S3). Some of the key quality parameters such as peptide and PSM confidence, *q*-value, and concatenated rank by server were found to be high (Supplementary Figure S4).

### Correlation of mRNA and Protein Data

**FIGURE 2:**
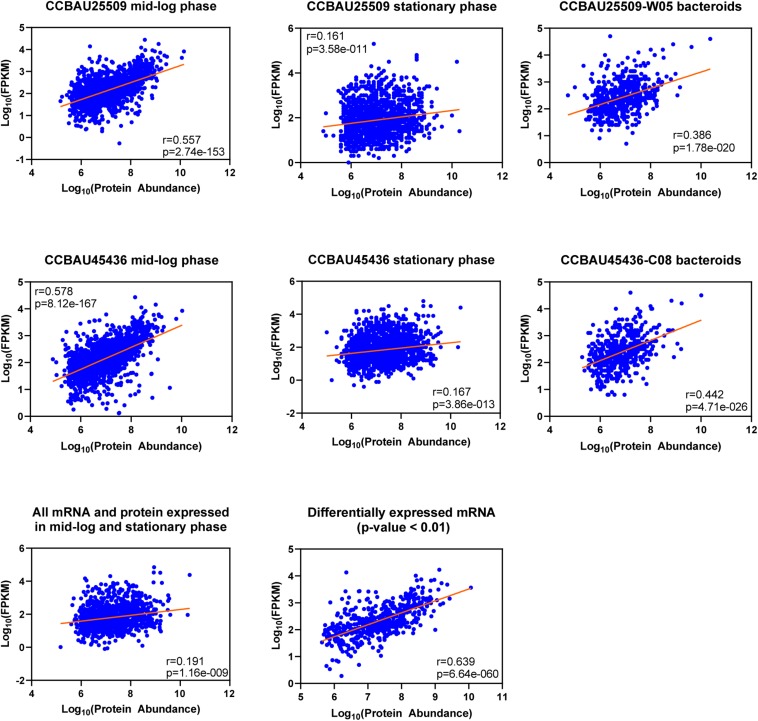
Overall correlation between the levels of label-free quantified (LFQ) proteins and mRNAs at different growth stages of *S. fredii* CCBAU25509 and CCBAU45436. The figures were drawn based on Log_10_ abundance values of proteome and transcriptome datasets. Averaged abundance of proteins of three biological replicates were used. W05: a *G. soja* accession; C08: a *G. max* accession. Pearson’s correlations were calculated using GraphPad Prism 8.2.1 at 95% confidence interval.

In the next step we compared the LFQ proteome data to RNA−sequencing data that have been recorded previously under the same growth stages by us (Jiao et al., 2018). The protein/mRNA−pairs from CCBAU25509 and CCBAU45436 during mid-log phase (CCBAU25509 vs. CCBAU45436: 1,869 vs. 1,863), stationary phase (1,665 vs. 1,871), and in bacteroids (527 vs. 509) were available to build correlations (Figure 2 and Supplementary Table S5). Among those pairs, proteins were quantified for all replicates. In mid-log phase, the correlation between LFQ protein abundance and mRNA expression levels was relatively high (*r* = 0.557 and 0.578 for CCBAU45436 and CCBAU25509, respectively), which is in the same range as the protein−mRNA correlation coefficient previously published for another Gram-negative bacterium (Kwon et al., 2014; Erdmann et al., 2018). A positive but weak correlation (*r* = 0.161 and 0.167) between LFQ protein and mRNA expression was observed during stationary phase. In bacteroids, the correlation between LFQ protein and mRNA expression is moderate (*r* = 0.386 and 0.442 for CCBAU25509 and CCBAU45436, respectively). These results are consistent with a general observation that the correlation between protein and mRNA levels is dependent on cell cycle and growth stage (Liu et al., 2016).

We also investigated the correlation between differentially expressed mRNA and their encoded proteins between mid-log phase and stationary phase in both strains combined. The correlation of differentially expressed mRNA/protein is much higher (*r* = 0.639) compared to the correlation of total expressed mRNA/protein (*r* = 0.191), indicating that gene expression with more dynamic changes may have higher mRNA-to-protein correlations (Erdmann et al., 2018). Since the number of expressed genes in bacteroids were much lower, we did not include them in this analysis.

### Secretome-Wide Nops Detection in CCBAU25509 and CCBAU45436

To investigate host-specific differences between the CCBAU25509 and CCBAU45436 strains, we performed a secretome analysis to identify Nops. We used genistein to induce the production of Nops as suggested by previous researches (Jiménez-Guerrero et al., 2017). Seven out of eight known Nops (NopA, NopB, NopC, NopD, NopI, NopL, NopP) were detected from the extracellular medium of both CCBAU45436 and CCBAU25509 using the Orbitrap system (Table 1). On the other hand, NopX was missing from the CCBAU25509 secretome, despite that the intact *nopX* gene was present in the CCBAU25509 genome with the same coding sequence as that in CCBAU45436. We also identified some other proteins in the secretome such as a 10- and a 60-kDa chaperonin, ATP-binding cassette (ABC) transporters, translation initiation factors, 50S ribosomal proteins, 30S ribosomal proteins, ATP-binding proteins, succinyl-CoA synthetase, outer-membrane lipoproteins, periplasmic dipeptide transport proteins and TolB protein precursor (Supplementary Table S6). The presence of ribosomal proteins and some other cytoplasmic proteins in the secretome could be the result of a non-classical (leaderless) mode of secretion described previously (Bendtsen et al., 2005). All the identified Nops and other secreted proteins had *q*-values < 0.01 (Table 1 and Supplementary Table S6).

**TABLE 1:** Orbitrap-based detection of Nops in CCBAU45436 and CCBAU25509 in response to genistein induction.

			**Coverage**			**# Unique**			
**Nops**	**Locus_tag**	***q*-Value**	**[%]**	**# Peptides**	**# PSMs**	**peptides**	**# AAs**	**MW [kDa]**	**calc. pI**
**CCBAU45436**									
NopA (pilus component)	AB395_RS31590	0	79	4	54	4	71	7	9.54
NopB (pilus component)	AB395_RS 31660	0	51	7	12	7	164	16.9	7.44
NopC (effector)	AB395_RS31595	0	15	1	1	1	98	9.8	4.81
NopD (effector)	AB395_RS30201	0	3	2	2	2	1490	159.6	5.31
NopI effector)	AB395_RS29941	0	11	2	2	2	285	31.7	5.05
NopL (effector)	AB395_RS31705	0	4	1	1	1	338	37.1	5.26
NopP (effector)	AB395_RS31605	0	14	3	4	3	270	31.1	4.96
NopX (pilus component)	AB395_RS31670	0	6	3	4	3	596	64	5.55
**CCBAU25509**									
NopA (pilus component)	AOX55_RS33065	0	93	8	81	8	71	7	9.54
NopB (pilus component)	AOX55_RS33135	0	66	8	11	8	164	16.9	7.44
NopC (effector)	AOX55_RS33070	0	15	1	1	1	98	9.8	4.81
NopD (effector)	AOX55_RS31761	0	2	1	1	1	1490	159.6	5.31
NopI (effector)	AOX55_RS31495	0	11	2	2	2	285	31.7	5.05
NopL (effector)	AOX55_RS33180	0	9	2	3	2	338	37.1	5.26
NopP (effector)	AOX55_RS33080	0	17	3	3	3	270	31.1	4.96

*PSMs, peptide spectrum matches; AAs, amino acids; MW, molecular weight; pI, isoelectric point.*

In *S. fredii* CCBAU25509 and CCBAU45436, all eight *nop* genes are located on the symbiotic plasmid (Figure 1B), as reported in other *S. fredii* strains (Schmeisser et al., 2009; Schuldes et al., 2012; Weidner et al., 2012; Jiao et al., 2018; Dang et al., 2019). We collected the transcriptomic data of each *nop* gene from our previous work (Jiao et al., 2018) to evaluate their expression patterns during the mid-log and stationary phases without genistein induction, and in the nodules of *G. max* (C08) and *G. soja* (W05) (Supplementary Figure S5). Except *nopX*, all the other *nop* genes showed high expressions at the stationary phase. Higher expressions of all eight *nop* genes were found in the C08 nodules compared to the W05 nodules. Since the NopP and NopI proteins may play a role in host specificity (see below), the transcriptomic data of the *nopP* and *nopI* genes were confirmed with RT-qPCR (Supplementary Figure S6).

### Genome-Wide *nop* Gene Distributions in *Sinorhizobium* and Closely Related Genera

**FIGURE 3:**
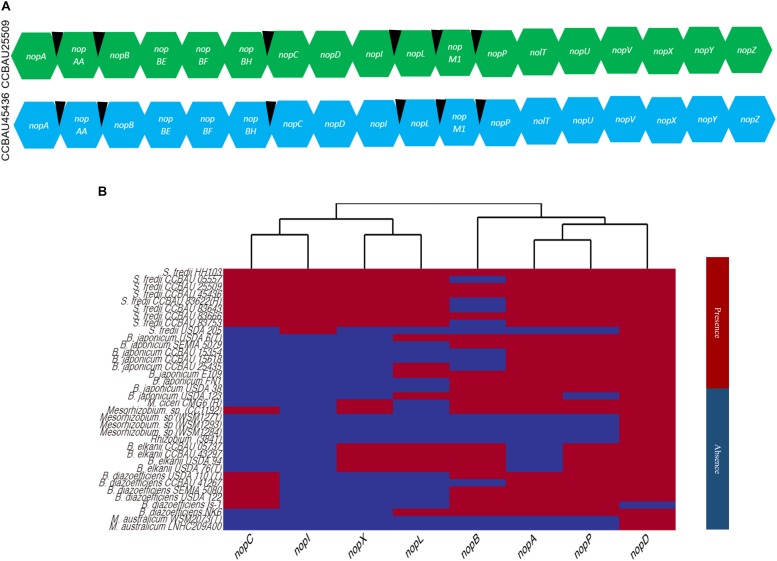
Known and putative *nop* genes identified in *S. fredii* CCBAU25509 and CCBAU45436 and the distribution of eight *nop* genes in 37 rhizobial strains. **(A)** Known and putative *nop* genes identified in the genomes of both *S. fredii* strains. Black wedges indicate the presence of *tts* boxes in the upstream regions of corresponding genes. **(B)** Cladogram showing the distribution of eight well-characterized *nop* genes in the genomes of 43 rhizobial strains belonging to four genera. Red and blue color indicate the presence or absence of a specific *nop*, respectively, in a particular genome.

Using the previously reported *nop* gene sequences from the strains, NGR234, USDA257 and USDA207, 18 known or putative *nop* genes (*nopA*, *nopAA*, *nopB*, *nopBE*, *nopBF*, *nopBH*, *nopC*, *nopD*, *nopL*, *nopI*, *nopP*, *nopM1*, *nolT*, *nopU*, *nopV*, *nopX*, and *nopZ*) were identified in CCBAU25509 and CCBAU45436 (Figure 3A and Supplementary Table S7). Six *tts* boxes were also identified in the upstream regions of six *nop* genes (*nopAA*, *nopM1*, *nopC*, *nopB*, *nopP*, and *nopL*) (Supplementary Table S7). In CCBAU25509, a membrane-anchored protein-coding gene and an *Fnr*-type transcriptional regulator were also found to be eventually regulated by *tts* boxes (Supplementary Table S7). In CCBAU45436, the *flavin mononucleotide* (*FMN*) *reductase* gene and a hypothetical protein-coding gene were found to have a *tts* box upstream of their respective coding sequences (Supplementary Table S7).

To get a better idea of the evolution of the Nops we detected in the extracellular medium upon flavonoid induction, we next investigated the distribution of these *nop* genes in *Sinorhizobium* and other rhizobia.

We downloaded 56 annotated and assembled genomes of the genera *Sinorhizobium*, *Mesorhizobium*, *Bradyrhizobium*, and *Rhizobium* (Supplementary Table S1). The phylogenetic tree built based on the 16S rRNA sequences resulted in two major clades. Clade I was made up of *Mesorhizobium*, *Sinorhizobium*, and *Rhizobium* while clade II was made up of *Bradyrhizobium*. In general, the genus *Sinorhizobium* was closer to the genus *Rhizobium* than to the genus *Mesorhizobium* (Supplementary Figure S7).

We searched for the *nop* genes that encode for the eight Nops discussed above in all 56 genomes and generated a heat map based on the presence or absence of each *nop* gene in all four genera (Figure 3). Out of 56 selected genomes, only 36 genomes yielded position hits and their genomic locations and sequences are listed in Supplementary Table S7. We did not find any hit for a *nop* gene sequence from all selected genomes belonging *S. medicae* and *S. meliloti* strains. Overall, a lineage-specific pattern was observed among strains and species for the presence or absence of these *nop* genes (Figure 3). As expected, the observed amino acid sequence similarities of encoded Nops were found to be higher between species than between genera for a particular Nop (Supplementary Figure S8).

NopD, which is a putative T3SS effector protein with a SUMO protease domain, was found in 35 of the 36 genomes containing *nop* genes, and was only missing in *B. diazoefficiens* (Is-1) strain (Rodrigues et al., 2007; Sánchez et al., 2012) (Supplementary Figure S8).

NopA (a pilus subunit protein) and NopL (a T3SS effector protein), were found in 25 and 17 genomes, respectively (Figure 3 and Supplementary Table S7). The predicted protein sequence similarity is close to 50% among all orthologs found, showing a high degree of conservation (Supplementary Figure S8). NopA, was present only in *S. fredii*, *B. japonicum*, *B. diazoefficiens* and two strains of *Mesorhizobium* (Figure 3). Another T3SS pilus component protein, NopB, was observed only in *S. fredii*, *B. elkanii*, *B. japonicum*, and *M. ciceri*.

NopP (a T3SS effector protein), which determines host specificity in *Bradyrhizobium* (Sugawara et al., 2018), was only found in *S. fredii*, *B. japonicum*, *B. elkanii*, *B. diazoefficiens*, *M. ciceri*, and *Mesorhizobium* sp. (CC1192). The major translocator protein, NopX, was found only in *B. elkanii, M. ciceri*, *Mesorhizobium* sp. (CC1192) and *S. fredii* strains (Figure 3).

The identified sequences of NopX in *B. elkanii* and *S. fredii* strains showed a high sequence similarity > 75% (Supplementary Figure S8). A high sequence similarity (>75%) was also observed among the Nops within the genus *Bradyrhizobium*, but < 50% identity when these were compared to the NopP from *S. fredii*. The same pattern was observed for NopD (Supplementary Figure S8).

On the other hand, NopI (a T3SS effector protein) was particularly identified in *S. fredii* strains only and appeared to be species-specific (Figure 3). NopC (a T3SS effector protein) was only found in *S. fredii, B. diazoefficiens*, and *Mesorhizobium* sp. (CC1192).

### Host Restriction of *S. fredii* Is Dependent on the Allelic Types of the Soybean Rj2/Rfg1 Protein

**FIGURE 4:**
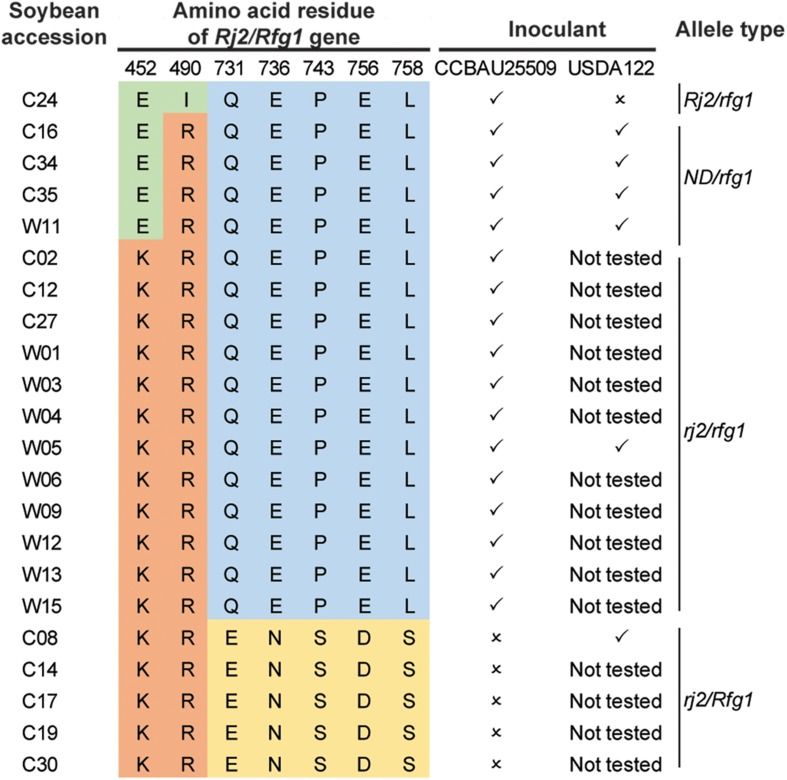
Genotypes and nodulation phenotypes of 22 soybean accessions. ✓: able to form nodules; ×: unable to form nodules. ND, not previously detected.

We selected 22 soybean accessions available in our laboratory (Lam et al., 2010) and performed PCR amplification and Sanger sequencing of the genomic regions encoding polymorphic amino acids (Yang et al., 2010) of the Rj2/Rfg1 protein. In addition to the three previously reported allelic types (Rj2/rfg1, rj2/rfg1, and rj2/Rfg1), a new allelic type with a previously unreported combination of amino acid polymorphism in the Rj2 region (E452, R490) was identified (Figure 4). All 22 soybean accessions were inoculated with the *S. fredii* strain CCBAU25509 and selected accessions were inoculated with the *B. diazoefficiens* strain USDA122 to characterize their nodulation phenotype (Figure 4). As expected, the Rj2/rfg1 and rj2/Rfg1 types exhibited restriction to *B. japonicum* strain USDA122 and *S. fredii* strain CCBAU25509 respectively, while the rj2/rfg1 allelic type imposed no restriction. The newly identified allelic type behaved like rj2. No Rj2/Rfg1 allelic type was found.

To further search for the allelic types of the Rj2(Rfg1) protein, we made use of the dataset from a large-scale re-sequencing project (Zhou et al., 2015). After removing accessions with ambiguous SNPs (either missing or heterozygous), 147 accessions showing clear allele types remained (Supplementary Table S8). Combining all available information, we found that the Rj2/rfg1 allele was the rarest while most of the accessions carried either the rj2/Rfg1 or the rj2/rfg1 allele.

### T3SS in *S. fredii* Is Essential for Host Restriction

**FIGURE 5:**
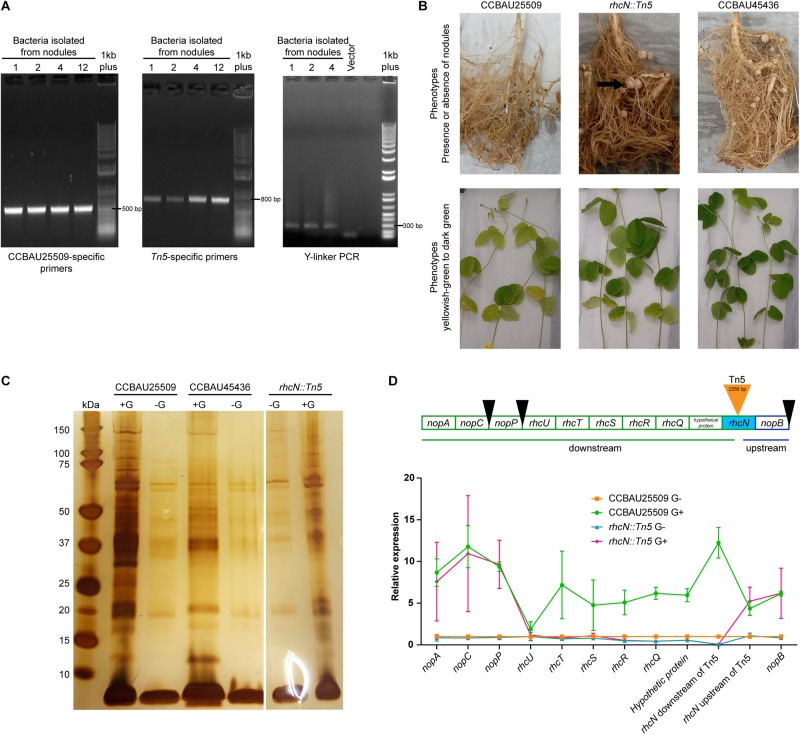
Characterization of the *rhcN:Tn5* mutant. **(A)** PCR was performed to confirm that the *rhcN:Tn5* mutant originated from CCBAU25509 (using CCBAU25509 primers) and contains a Tn5 insertion (using Tn5 primers and Y-linker PCR). Numbers 1, 2, 4, and 12 are rhizobia isolated from independent nodules. However, they were probably the same insertional event since the junction sequences were identical. Last lane on each gel is the 1 kb Plus DNA Ladder (Invitrogen). **(B)** Performance of the *rhcN:Tn5* mutant inoculated onto the soybean accession C08 that carries the *Rfg1* allele. Formation of root nodules (indicated by black arrows) and the leaf color (pale green or dark green) when the plants were grown under nitrogen-limiting conditions were recorded for the *rhcN:Tn5* mutant, the non-compatible parent strain CCBAU25509, and the compatible strain CCBAU45436 28 days post-inoculation. **(C)** Silver staining visualization of the NuPAGE 4–12% bis-tris gel of the precipitated extracellular proteins from CCBAU25509, CCBAU45436 and the *rhcN:Tn5* mutant with and without genistein (G) induction. **(D)** Upper panel: genes upstream and downstream of the *rhcN* operon. Black wedges indicate the upstream *tts* box sequences. Lower panel: relative expression patterns of these genes in CCBAU25509 and in the *rhcN:Tn5* mutant with and without genistein (G). *GyrA* was used as the reference gene. Each data point represents the mean of three independent biological replicates. Error bar: standard deviation.

By taking advantage of the incompatibility of CCBAU25509 with the soybean host C08 (rj2/Rfg1), we performed Tn5 insertion mutagenesis in CCBAU25509 to select for mutants that could successfully nodulate C08. We generated a mixture of > 1.27 × 10^6^ Tn5-insertion mutants by bi-parental mating of CCBAU25509 with *E. coli* S17-1 λpir harboring pUTmini-Tn5 km (Biomedal Life Science, Spain). The mixture of Tn5 mutants of CCBAU25509 was then used to inoculate 300 seedlings of C08. Four well-formed nodules resulted and were harvested at 28 days post-inoculation. Isolates from these four nodules were further confirmed by re-inoculating them into C08, of which three were able to form effective nodules after re-inoculation. The presence of the Tn5 insertion in these isolates were confirmed by PCR with strain-specific primers and Tn5-specific primers, and by Y-linker PCR and sequencing (Figure 5A). Results showed that these three isolates were likely the result of a single event having the mini-Tn5 transposon of 2,356 bp inserted in the middle of a coding region resembling an ortholog of *rhcN* (pSF25509a: 342,898.342,897 bp). RhcN was previously identified in the *S. fredii* strain NGR234 as a putative ATP synthase involved in the secretion of Nops through the lumen of the T3SS pilus (Staehelin and Krishnan, 2015).

The *rhcN:Tn5* insertion in CCBAU25509 removed host incompatibility with C08, which, when inoculated with this mutant, were successfully nodulated and exhibited a higher chlorophyll content (an indicator of nitrogen contents *in planta*) under nitrogen-limiting conditions (Figure 5B and Table 2).

**TABLE 2:** Performance of soybean accession C08 carrying the *Rfg1* allele when inoculated with CCBAU25509, the *rhcN:Tn5* mutant of CCBAU25509, or CCBAU45436 28 days post-inoculation.

	**Number of nodules**	**Nodule fresh weight**	**Nodule size**	**Leaf chlorophyll**
**Inoculant**	**(per C08 seedling)**	**(mg/nodule)**	**(mm/nodule)**	**(μg/mL)**
Uninoculated control	–	–	–	496.0 ± 50.4*
CCBAU45436	51.9 ± 2.71***	10.9 ± 0.58**	2.65 ± 0.07**	956.3 ± 41.7***
CCBAU25509	–	–	–	505.5 ± 33.1**
*rhcN:Tn5* mutant	11.7 ± 1.53	20.0 ± 2.42	3.23 ± 0.16	648.4 ± 32.3

Data represent mean ± standard error. All statistical comparisons were made against the accession C08 seedlings inoculated with the *rhcN:Tn5* mutant. Asterisks indicate statistical significance: ^*^p < 0.05; **p < 0.01; ***p < 0.001.

To investigate the impact of the *rhcN:Tn5* mutation on the secretion of Nops, we repeated the secretome experiment with the mutant strain. Secreted proteins that are not related to T3SS, such as the 60-kDa chaperonin, translation initiation factor IF-2, outer-membrane lipoproteins, etc., were still present in the secretome (Supplementary Table S6). On the other hand, no Nops, except NopA, were found in the secretome upon genistein induction (Figure 5C). Since the absence of Nops in the *rhcN:Tn5* mutant improved the host compatibility, it could be inferred that Nops play a role in the incompatibility of CCBAU25509 with some soybean cultivars. Moreover, NopX, which was not detected in the secretome of the wild type CCBAU25509 (Table 1), was unlikely to have contributed to host incompatibility.

We then investigated the expression patterns of other genes in the same operon of *rhcN*, including the upstream *nopB* and other downstream genes: a hypothetical protein-coding gene, *rhcQ*, *rhcR*, *rhcS*, *rhcT*, *rhcU*, *nopP*, *nopC*, and *nopA*, in the presence or absence of genistein, using the gene expression patterns of the wild type strain CCBAU25509 as the control (Figure 5D). Without genistein, the expression levels of the above genes between CCBAU25509 and the *rhcN:Tn5* mutant were comparable, except for *rhcQ*, *rhcR* and a hypothetical protein-coding gene, located immediately downstream of the Tn5 insertion, which had lower expressions in the *rhcN* mutant. In the presence of genistein, all the above-mentioned genes were up-regulated in the wild type strain of CCBAU25509. However, in the *rhcN:Tn5* mutant, only *nopB* (located upstream of the Tn5 insertion site) and *nopP*, *nopC*, and *nopA* (far downstream of the insertion site) were induced by genistein. Their degree of induction by genistein in the *rhcN:Tn5* mutant was comparable to that in the wild type CCBAU25509. In the *rhcN:Tn5* mutant, the lower expression of hypothetical protein-coding gene, *rhcQ*, *rhcR*, *rhcS*, *rhcT*, *rhcU* genes compared to higher expression in CCBAU25509 in response to genistein suggests that the nodulation phenotype in the *rhcN:Tn5* mutant may be due to the disruption of multiple components of the T3SS.

### Conservation of NopP and NopI Protein Sequences in Host-Restricted *S. fredii* Strain

The Rj2/Rfg1 protein is the soybean determinant restricting nodulation by some *B. japonicum*, *B. diazoefficiens*, and *S. fredii* strains (Yang et al., 2010; Fan et al., 2017). Polymorphisms of seven amino acid residues (E452K, I490R, Q731E, E736N, P743S, E756D, and R758S) were used to define three allelic groups of Rj2/Rfg1 (Yang et al., 2010; Fan et al., 2017). Rj2/rfg1 restricts some *B. japonicum* and *B. diazoefficiens* strains; and rj2/Rfg1 restricts some *S. fredii* strains. On the other hand, rj2/rfg1 allows most *B. japonicum*, *B. diazoefficiens*, and *S. fredii* to form nodules.

We compared the sequences of Nops between Rfg1-compatible and Rfg1-incompatible *S. fredii* strains, such as CCBAU45436 and CCBAU25509, respectively. Protein sequences of most Nops were either completely identical between the two strains (NopA, NopC, and NopL) or showed no host-specific sequence conservation (NopB and NopX) (Supplementary Figure S9). The exceptions are the host-specific sequence conservation of NopP and NopI, which may be the culprits of host incompatibility. All amino acid substitutions in NopI and NopP were confirmed by PCR-based amplification, cloning and sequencing.

**FIGURE 6:**
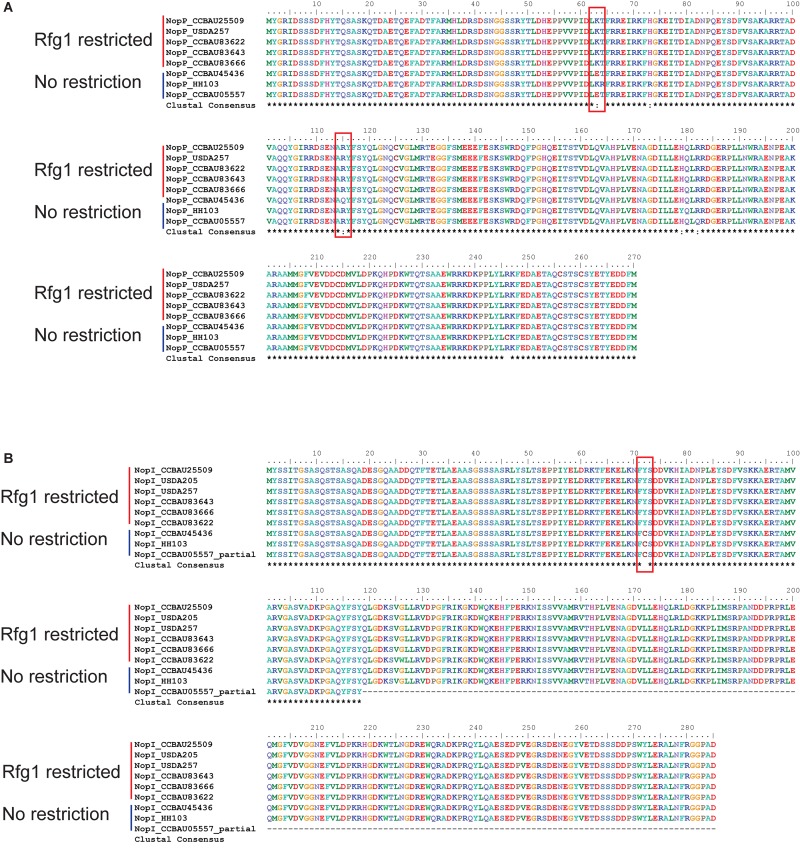
NopI and NopP protein sequence alignments between *Rfg1*-compatible and incompatible *S. fredii* strains. Shown host compatibility is based on observed phenotypes in previous publications (Yang et al., 2010; Jiménez-Guerrero et al., 2017; Jiao et al., 2018; Temprano-Vera et al., 2018). **(A)** NopP alignment showing K63E and R115Q amino acid substitutions. **(B)** NopI alignment showing a Y72C amino acid substitution.

The NopP from *Bradyrhizobium* was reported to be a bacterial determinant of host incompatibility in soybean expressing the Rj2 protein, although no direct interaction between NopP and Rj2 has been found (Sugawara et al., 2018). In soybean expressing the *Rfg1* allele, the NopP sequences in the restrictive *S. fredii* strains are invariable while there are some non-synonymous changes in the non-restrictive strains (Figure 6). We also observed two amino acid substitutions (K63E and R115Q) in NopP in the non-restrictive *S. fredii* CCBAU45436 compared to the restrictive CCBAU25509 (Figure 6A). One of the amino acids in the NopP of CCBAU25509 (K63) was observed at the same position as one previously reported in the NopP from *B. diazoefficiens* (USDA 122) (R60) and is proposed to be a key residue for *Rj2*-mediated incompatibility in soybean (Sugawara et al., 2018).

NopI is a *S. fredii*-specific Nop which exhibits 48% sequence identity to NopP in the strain HH103 (Jiménez-Guerrero et al., 2017). The alignment of NopI showed an amino acid substitution (Y72C) in CCBAU45436 compared to CCBAU25509 (Figure 6B). We extracted the protein sequences of Nops from *S. fredii* strains with known host specificity for sequence alignments. NopI proteins are clearly divided into two groups among restrictive and non-restrictive *S. fredii* strains (Figure 6B).

### NopP Played Essential Roles in Determining Host Compatibility

**FIGURE 7:**
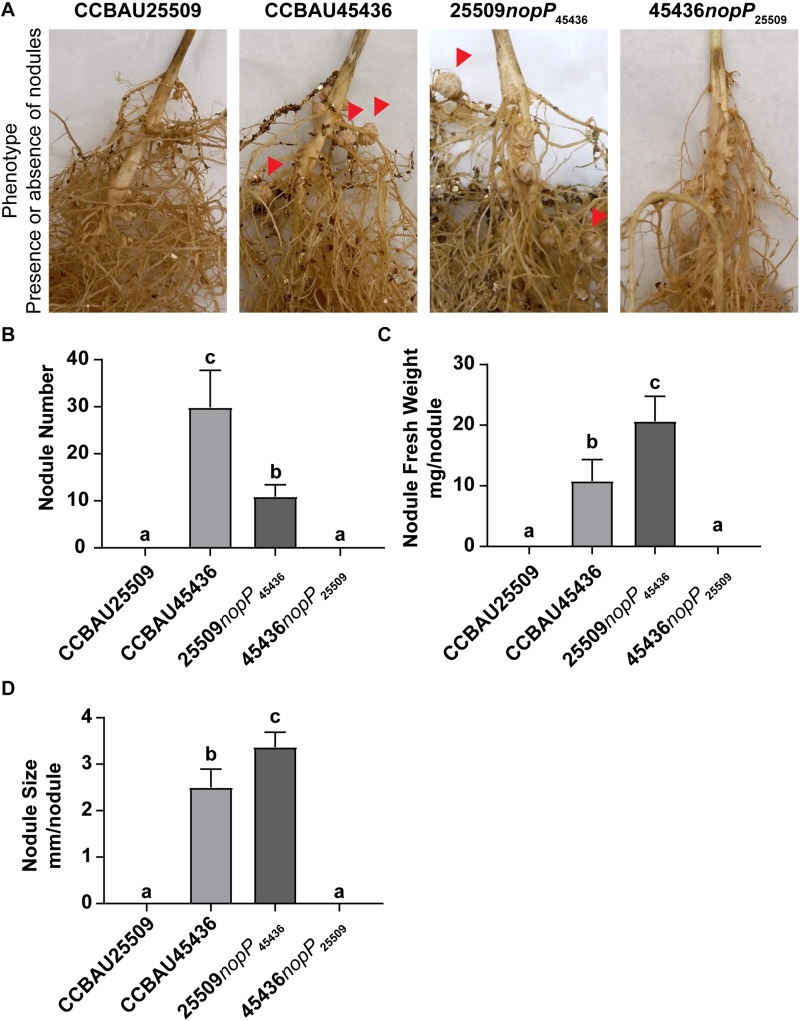
Nodulation phenotype of *S. fredii* strains carrying different NopP alleles. **(A)** Root of *G. max* C08 inoculated with wild type *S. fredii* CCBAU25509, wild type *S. fredii* CCBAU45436, mutant of CCBAU25509 carrying NopP from CCBAU45436 (25509*nopP*_45436_), and mutant of CCBAU45436 carrying NopP from CCBAU25509 (45436*nopP*_25509_). C08 carries the *rj2/Rfg1* allele. Red arrows indicate some effective nodules formed on the roots. **(B)** Nodule number, **(C)** Nodule fresh weight and **(D)** Nodule size of the nodules harvested from the inoculated roots 28 days post-inoculation. *N* = 8. Error bar: standard deviation. Different letters above the bars indicate the results are statistically different at *p* < 0.05. The experiment has been repeated twice with consistent results (Supplementary Figure S10).

Here we compared the nodulation phenotypes among wild type CCBAU25509, wild type CCBAU45436, CCBAU25509 carrying only NopP from CCBAU45436 (25509*nopP*_45436_), and CCBAU45436 carrying only NopP from CCBAU25509 (45436*nopP*_25509_). The bacteria were inoculated into *G. max* C08 which carries the *rj2/Rfg1* allele. Our results showed that only those strains carrying NopP from CCBAU45436 (wild type CCBAU45436 and 25509*nopP*_45436_) were able to nodulate C08 while those carrying NopP from CCBAU25509 (wild type CCBAU25509 and 45436*nopP*_25509_) could not (Figure 7 and Supplementary Figure S10).

This finding led us to investigate if there is any direct interaction between Rfg1 and the two isoforms of sinorhizobial NopP using yeast two-hybrids (Supplementary Figure S11). The results did not demonstrate any direct interaction between Rfg1 and either forms of NopP. It is possible that NopP may bind to an unknown decoy in soybean which mediates the restriction functions of Rfg1.

## Discussion

One important environmental value of leguminous plants is their ability to form root nodules which fix atmospheric dinitrogen molecules by converting them to ammonia through symbiotic relationships with soil rhizobia (Dixon and Kahn, 2004). Because of this unique property, cultivation of soybean, a leguminous crop, has become an integral part of sustainable agriculture. For example, a recent modeling study predicts that large-scale replacement of monocropping maize with intercropping maize-soybean could reduce the amount of nitrogen fertilizer used and subsequently alleviate air pollution problems caused by the emission of nitrogen oxides as a result of the excessive use of nitrogenous fertilizers (Fung et al., 2019).

To better characterize these rhizobial strains and their symbiotic relationships with soybean, we have previously completed the full genomes and conducted comprehensive transcriptomic analyses of two *S. fredii* strains, CCBAU25509 and CCBAU45436, which exhibit differential host compatibility toward soybean carrying the *Rfg1* allele (Jiao et al., 2018). These two strains are descendants of a dominant sub-lineage in northeastern China (Temprano-Vera et al., 2018) and hence are representative strains.

To better characterize these two strains, we conducted high-throughput proteomic analyses to provide a unique view of proteins produced during mid-log phase, stationary phase, and in the bacteroid form. These data are a valuable resource for the study of *S. fredii* and symbiosis (Supplementary Tables S3, S4). The constructed circular proteome map with reference to its distribution within the genome suggests that the core genome has a higher priority in translation compared to plasmids (Figure 1B), which is consistent with previous research in *Bradyrhizobium* (Delmotte et al., 2014). These results are consistent with the findings in a previous proteome profiling experiment done in *S. melioti* (Sobrero et al., 2012; Berrabah et al., 2018). The most abundant protein species are chaperonins in all three growth stages, suggesting that they are the key proteins to sustain protein homeostasis (Wälti et al., 2017). The presence of phasin family proteins in all three growth stages of *S. fredii* indicates that they are multipurpose proteins which the cell always needs for regulating growth (Mezzina and Pettinari, 2016). The substantially expressed carbonic anhydrase protein in mid-log phase suggests that CO_2_ conversion to bicarbonate is necessary for growth in the free-living form (Merlin et al., 2003). The appearance of ABC transporter proteins, TCA cycle proteins, cold shock proteins, ferredoxin family proteins, nitrogen fixation and regulatory proteins, phosphate transport proteins, outer membrane lipoproteins, as well as transcriptional regulator proteins in bacteroids collected from nodules suggest that these proteins are important pillars of survival and nitrogen fixation in the symbiotic form.

In this study, we have established a new protocol to study the secretome by harnessing the power of Orbitrap. ABC transporters, ATP-binding protein, outer membrane lipoproteins, periplasmic dipeptide transport proteins, and TolB protein precursors were found in the extracellular medium of bacterial culture (Supplementary Table S6), suggesting possible roles in symbiosis (Broughton et al., 2000).

In addition to other secreted proteins, we also successfully detected eight previously reported Nops (NopA, NopB, NopC, NopD, NopI, NopL, NopP, and NopX) in the extracellular medium of bacterial culture upon genistein induction (Table 1). NopA, NopB, and NopX are components of the injectosome while the others, NopC, NopD, NopI, NopL and NopP, are possibly effector proteins secreted by T3SS into the host cytoplasm. These results are supported by previous studies performed on individual Nops in other *Sinorhizobium* strains (Krishnan et al., 2003; Marie et al., 2003; Lorio et al., 2004; Rodrigues et al., 2007; Zhang et al., 2011; Kim and Krishnan, 2014; Jiménez-Guerrero et al., 2015, 2017; Ge et al., 2016; Sugawara et al., 2018).

To analyze the genome-wide *nop* gene distribution in *Sinorhizobium* and other closely related genera, we downloaded 56 genomes covering *Sinorhizobium*, *Mesorhizobium*, *Rhizobium*, and *Bradyrhizobium*. Phylogenetic analyses using 16S rRNA differentiated *Bradyrhizobium* as a separate clade from the other three genera. Indeed, it was estimated that *Bradyrhizobium* had separated from the other clade before the emergence of legumes (Tian et al., 2012).

Despite the large evolutionary distance of *Bradyrhizobium* from the other three genera, on average there is a sequence similarity of > 50% among the eight Nops across all four genera (Supplementary Figure S8), suggesting a mutualistic co-evolution of T3SS effector proteins (Kimbrel et al., 2013).

Overall, the observed lineage-specific patterns of Nops (NopA, NopB, NopC, NopD, NopI, NopL, NopP, and NopX) distribution across all four genera indicate that they are distantly located in the symbiosis islands of their respective genomes, which cannot be explained solely by horizontal gene transfer or high genome-wide diversity (Kaneko et al., 2002; Kimbrel et al., 2013; Hungria et al., 2015) (Figure 3). In response to genistein induction, the observed expressions of Nops in the form of FPKM values are consistent with the previously published expression patterns of NopA, NopB, NopX, and NopP in *S. fredii* HH103 (Pérez-Montaño et al., 2016) (Supplementary Figure S5).

The presence of NopD in all four genera suggests that this effector protein in T3SS remains conserved during the adaptation to various hosts and geographic regions (Figure 3) (Kimbrel et al., 2013). NopI is absent in *Bradyrhizobium* (Figure 3) while some other Nops, such as NopE, NopH, pgl and NopF, are secreted by *Bradyrhizobium* but not by *Sinorhizobium* (Hempel et al., 2009). Although both *Bradyrhizobium* and *Sinorhizobium* strains can nodulate the same soybean host, they probably acquired divergent machineries for symbiosis that are functionally similar, independently of each other (Tian et al., 2012). The absence of NopX in *B. diazoefficiens* and *B. japonicum* suggests that, during divergence from *B. elkanii*, they might have lost this gene (Figure 3).

Between CCBAU25509 and CCBAU45436, there are seven common genistein-inducible Nops while NopX alone is undetectable in the extracellular medium of CCBAU25509 (Table 1). Although the *nopX* genes share an identical coding sequence in both CCBAU25509 and CCBAU45436, based on our transcriptome data (Supplementary Figure S5), it is likely that the expression of *nopX* gene in CCBAU25509 is turned off.

*Bradyrhizobium diazoefficiens* and *S. fredii* are restricted by some soybean hosts depending on the allelic type of the Rj2(Rfg1) protein. Extended analysis of soybean germplasm showed that there are three major allelic types: Rj2/rfg1, rj2/Rfg1, and rj2/rfg1, whereas Rj2/rfg1 seems to be a minor allelic type. CCBAU25509 is restricted by C08 which carries the *Rfg1* allele while CCBAU45436 is not. It is largely unknown how Rfg1 recognizes the incompatible strain and restricts its nodulation. To investigate the role of T3SS in the host restriction of CCBAU25509, we constructed a mutant in CCBAU25509 that carries an insertional mutation in the *rhcN* gene. This insertion resulted in the deactivation of several T3SS components in the same operon (Figure 5D). As a result, the *rhcN:Tn5* CCBAU25509 mutant could successfully nodulate the soybean accession that contains the *Rfg1* allele.

Since the removal of T3SS allows CCBAU25509 to bypass the restriction imposed by Rfg1, the absence of NopX in the CCBAU25509 secretome is unlikely to be the cause of host incompatibility. While the sequence of NopA is conserved between CCBAU25509 and CCBAU45436, and could still be detected in the secretome of the *rhcN:Tn5* CCBAU25509 mutant, the remaining six Nops were no longer detectable in the secretome of the mutant.

Interestingly, sequence alignments show that the protein sequences of NopP and NopI, but not the other Nops, are conserved in the *Rfg1*-restricted *S. fredii* strain (Figure 6 and Supplementary Figure S9). NopP and NopI are paralogs playing different roles in the nodulation of other rhizobial strains (Jiménez-Guerrero et al., 2017; Sugawara et al., 2018). The *nopP* of *B. diazoefficiens* was reported to genetically interact with *Rj2* in determining the host specificity (Sugawara et al., 2018), while knocking out the *nopI* of *S. fredii* HH103 reduced the nodule numbers (Jiménez-Guerrero et al., 2017).

The highly conserved amino acid substitutions in NopI and NopP of CCBAU25509 could be potential amino acid candidates for studying the differential host compatibility between these two strains (Figure 6). In *B. diazoefficiens* (USDA122), a mutation in the same position (residue 63 of NopP from *S. fredii* and residue 60 of NopP from *B. diazoefficiens*) in NopP changes the compatibility toward soybeans carrying an *Rj2* allele (Sugawara et al., 2018). However, *B. diazoefficiens* carrying the *Rj2*-incompatible NopP could still nodulate soybeans carrying the *Rfg1* allele (Yang et al., 2010).

The effects of NopP in *S. fredii* on legume nodulation has been widely studied in the previous decades. However, specific studies on the effects of NopP in soybean nodulation are few. It is reported that disruption in what *S. fredii* HH103 could lead to an increase in nodule number on the roots of its *Rfg1*-carrying compatible soybean host (López-Baena et al., 2009).

In this study, by swapping the NopP between CCBUA25509 and CCBAU45436, it showed that only the strains carrying *nopP*_45436_ could successfully nodulate C08 (Figure 7 and Supplementary Figure S10). Although the nodule number generated by 25509*nopP*_45436_ was lower than that of wild type CCBAU45436, the restriction barrier between C08 and CCBAU25509 was overcome. On the other hand, unlike wild type CCBAU45436, CCBAU45436*nopP*_25509_ showed a restrictive nodulation phenotype similar to wild type CCBAU25509. This suggests that NopP may serve as a determinant in nodulation restriction by soybeans expressing Rfg1, based on its amino acid sequence, by either permitting nodulation (NopP_45436_ with E63 and Q115) or by restricting nodulation (NopP_25509_ with K63 and R115). It is speculated that the NopP_25509_ may serve as one of the effectors recognized by the Rfg1-expressing hosts and trigger the host defense mechanism while NopP_45436_ may serve as an effector that inhibits or escapes the host defense response. These results suggest that, similar to the NopP in *Bradyrhizobium*, the NopP in *S. fredii* also plays a role in host nodulation restrictions, and the amino acids at positions 63 and 115 may serve as restriction determinants between *S. fredii* and Rfg1-type soybean hosts.

Our yeast two-hybrid assay failed to detect any direct interaction between NopP and Rfg1 *in vivo* in the heterologous system (Supplementary Figure S11). However, it was reported that many effectors do not directly interact with the R proteins, but instead trigger defense responses by binding to decoys (Büttner, 2016). Of course, we cannot rule out the possibility that post-translational modifications in the native organism may be required for the interaction (Skorpil et al., 2005; Sugawara et al., 2018).

In summary, we provided comprehensive proteomic and secretomic information on two model strains of *S. fredii* to facilitate further in-depth analyses of *S. fredii* and its symbiotic relationships with different soybean accessions.

## Data Availability Statement

The raw data supporting the conclusions of this manuscript will be made available by the authors, without undue reservation, to any qualified researcher.

## Author Contributions

H-ML coordinated this overall research strategy. HR and W-LC wrote the first draft of the manuscript. HR performed the Orbitrap-based proteomic and secretomic analyses. S-NT helped to optimize the LC gradient for secretome analysis. W-LC, W-TT, and L-YC identified and characterized the *rhcN:Tn5* CCBAU25509 mutant. K-SW, C-YL, and F-LW characterized the nodulation genotype and phenotype of soybean accessions. MX performed soybean re-sequencing data analysis. C-YL and W-LC performed yeast two-hybrid experiments. W-LC created and characterized the NopP mutants. M-WL and H-ML edited and proofread the final manuscript.

## Conflict of Interest

The authors declare that the research was conducted in the absence of any commercial or financial relationships that could be construed as a potential conflict of interest.

## Footnotes

1 https://cg.ezbiocloud.net

2 http://www.megasoftware.net/
