# Supplementary material for: High-Throughput Mass Spectrometric Analysis of the Whole Proteome and Secretome From Sinorhizobium fredii Strains CCBAU25509 and CCBAU45436
Source: Front Microbiol. 2019 Nov 12;10:2569. doi: 10.3389/fmicb.2019.02569 (PMC6865838; doi:10.3389/fmicb.2019.02569)
Supplement: Supplementary file 9 [file Presentation_1.PPTX]

## Slide 1
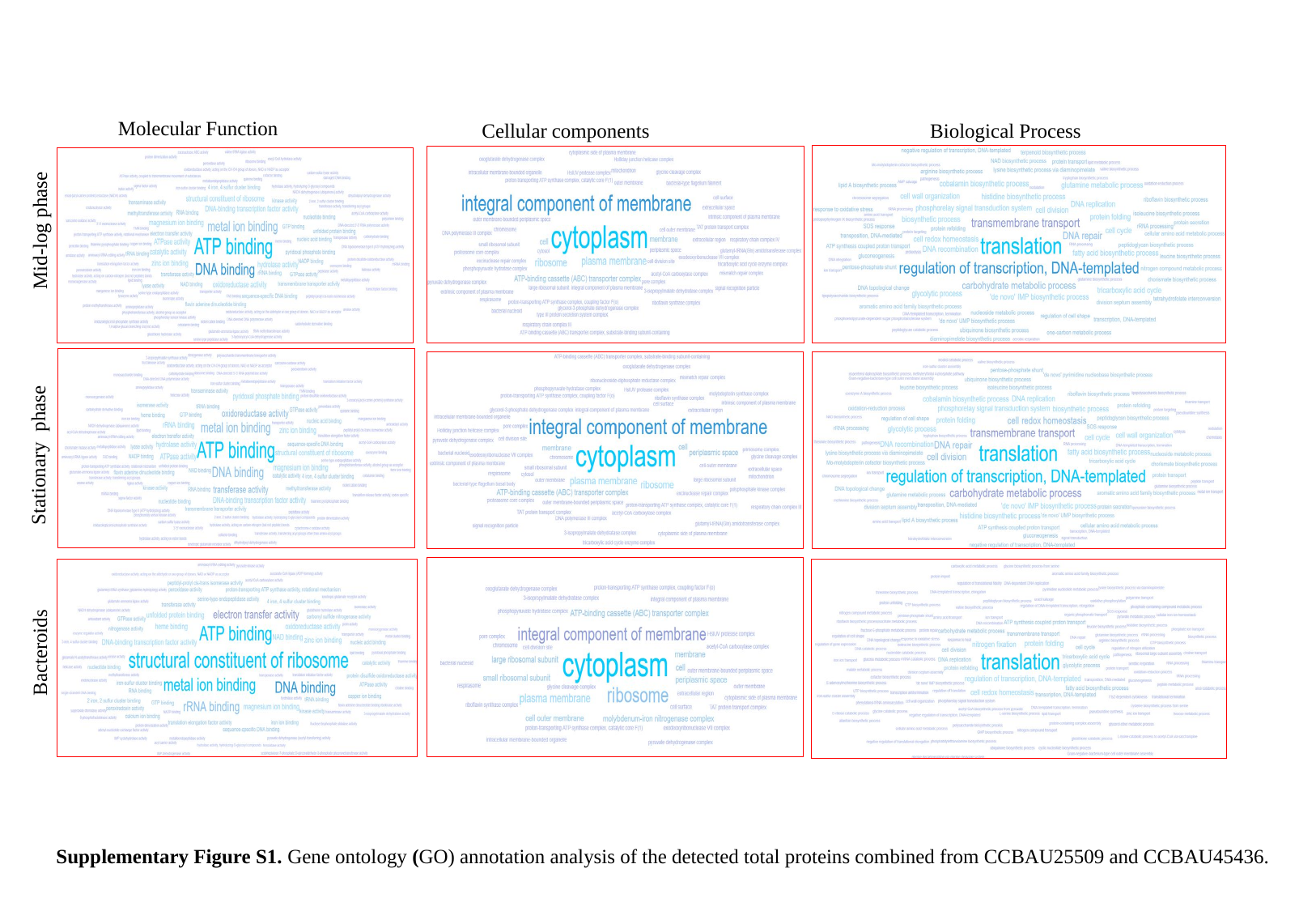

Molecular Function
Cellular components
Biological Process
Mid-log phase
Stationary phase
Bacteroids
Supplementary Figure S1. Gene ontology (GO) annotation analysis of the detected total proteins combined from CCBAU25509 and CCBAU45436.

## Slide 2
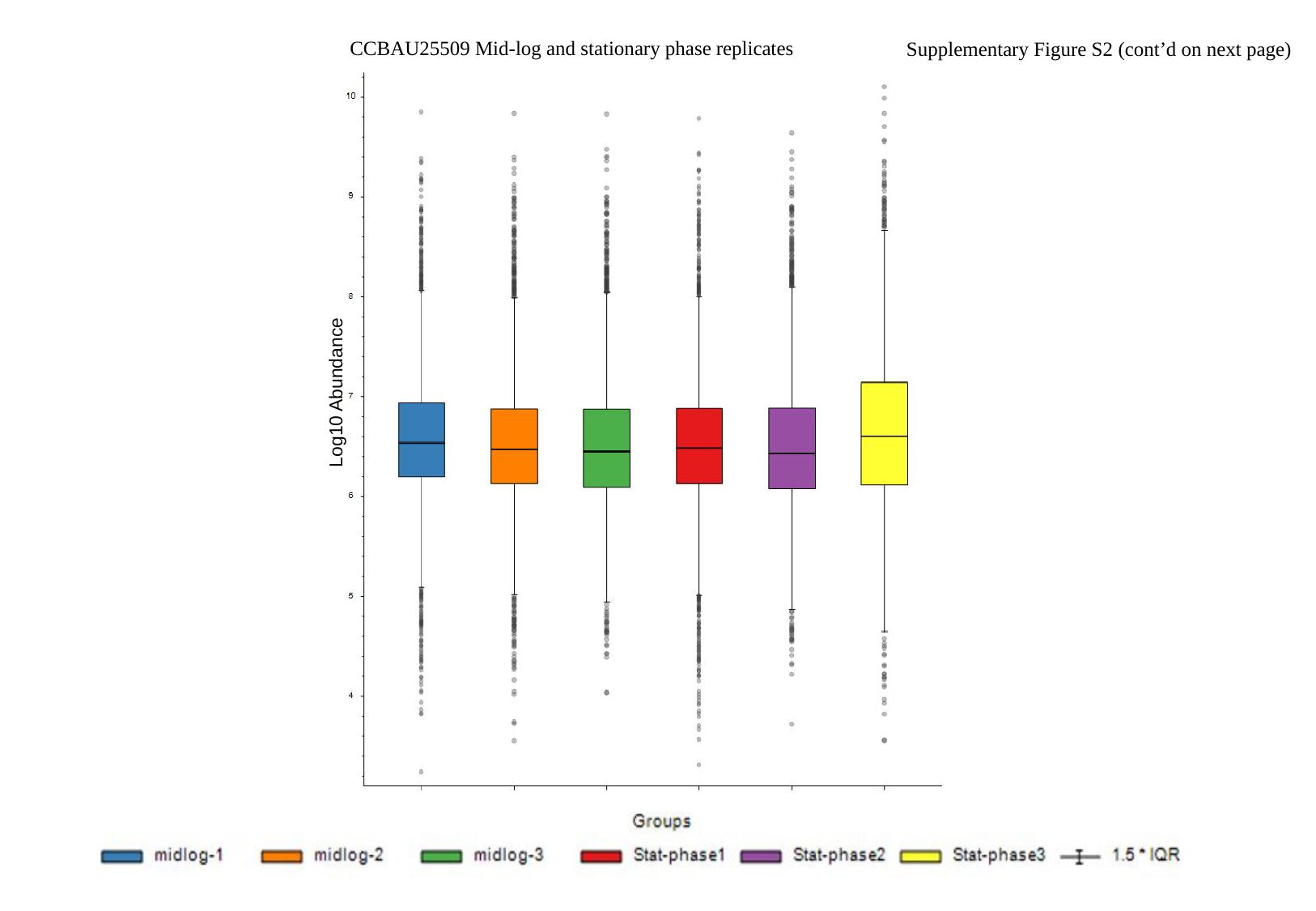

CCBAU25509 Mid-log and stationary phase replicates
Supplementary Figure S2 (cont’d on next page)
Log10 Abundance

## Slide 3
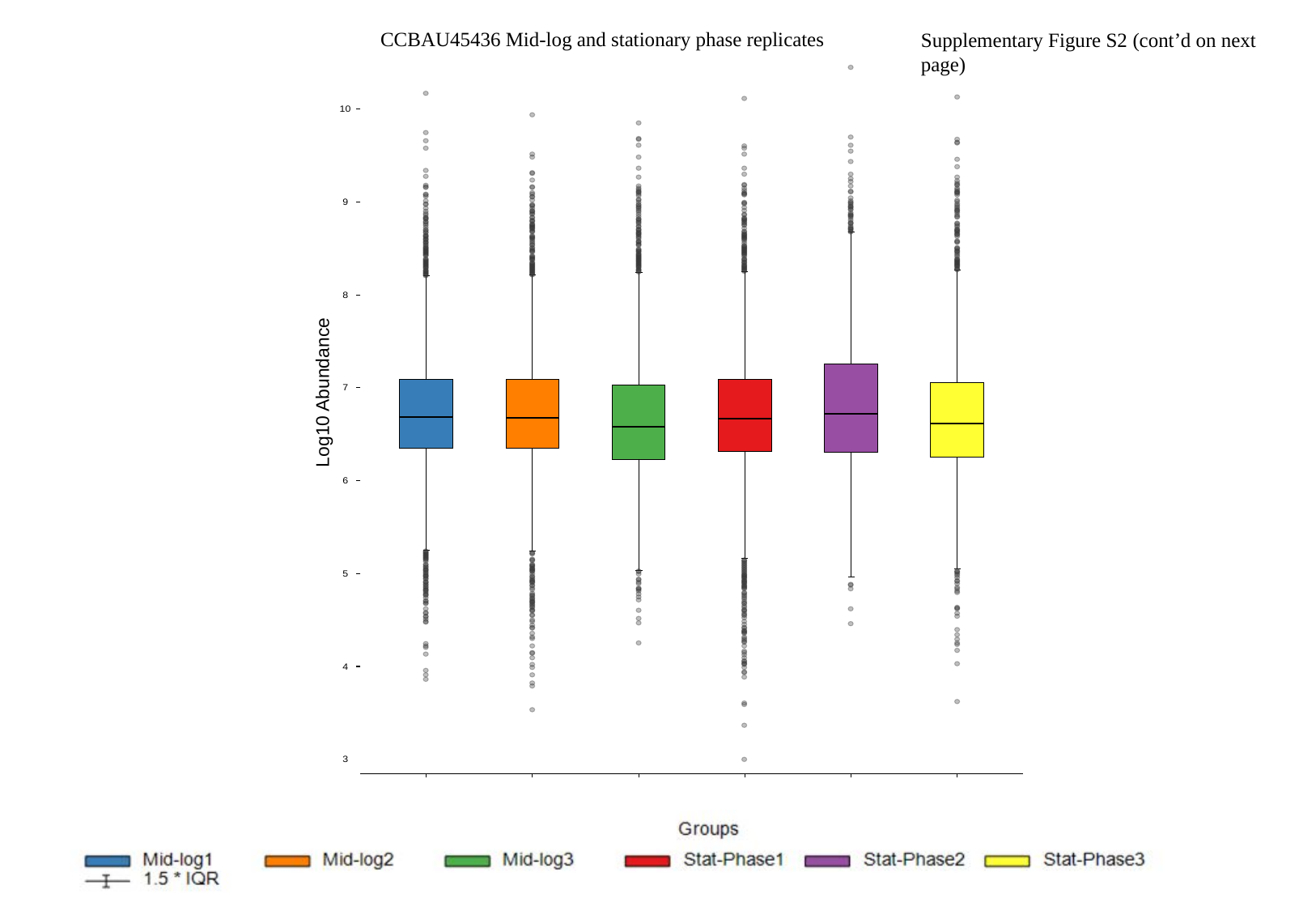

CCBAU45436 Mid-log and stationary phase replicates
Supplementary Figure S2 (cont’d on next page)
Log10 Abundance

## Slide 4
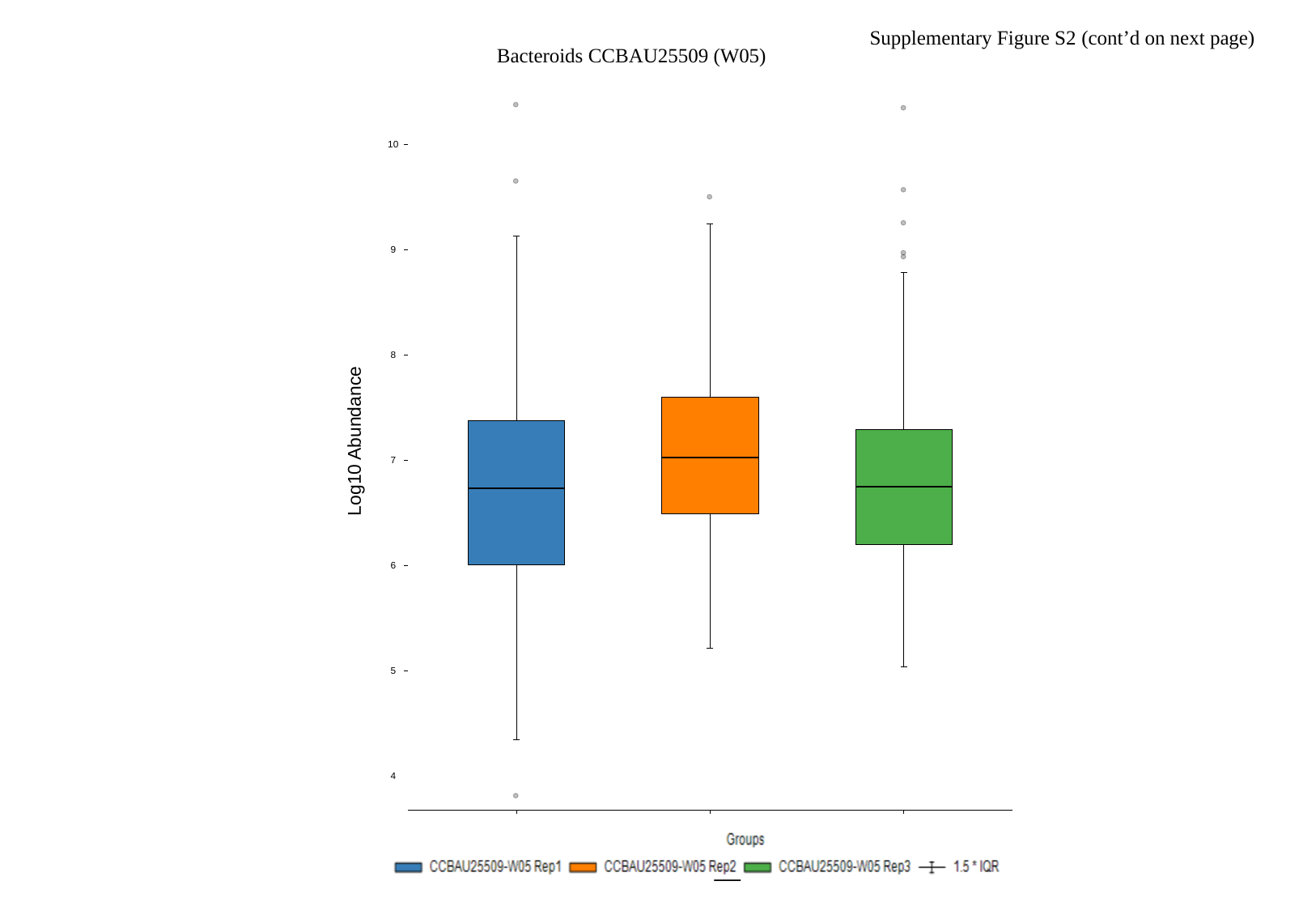

Supplementary Figure S2 (cont’d on next page)
Bacteroids CCBAU25509 (W05)
Log10 Abundance

## Slide 5
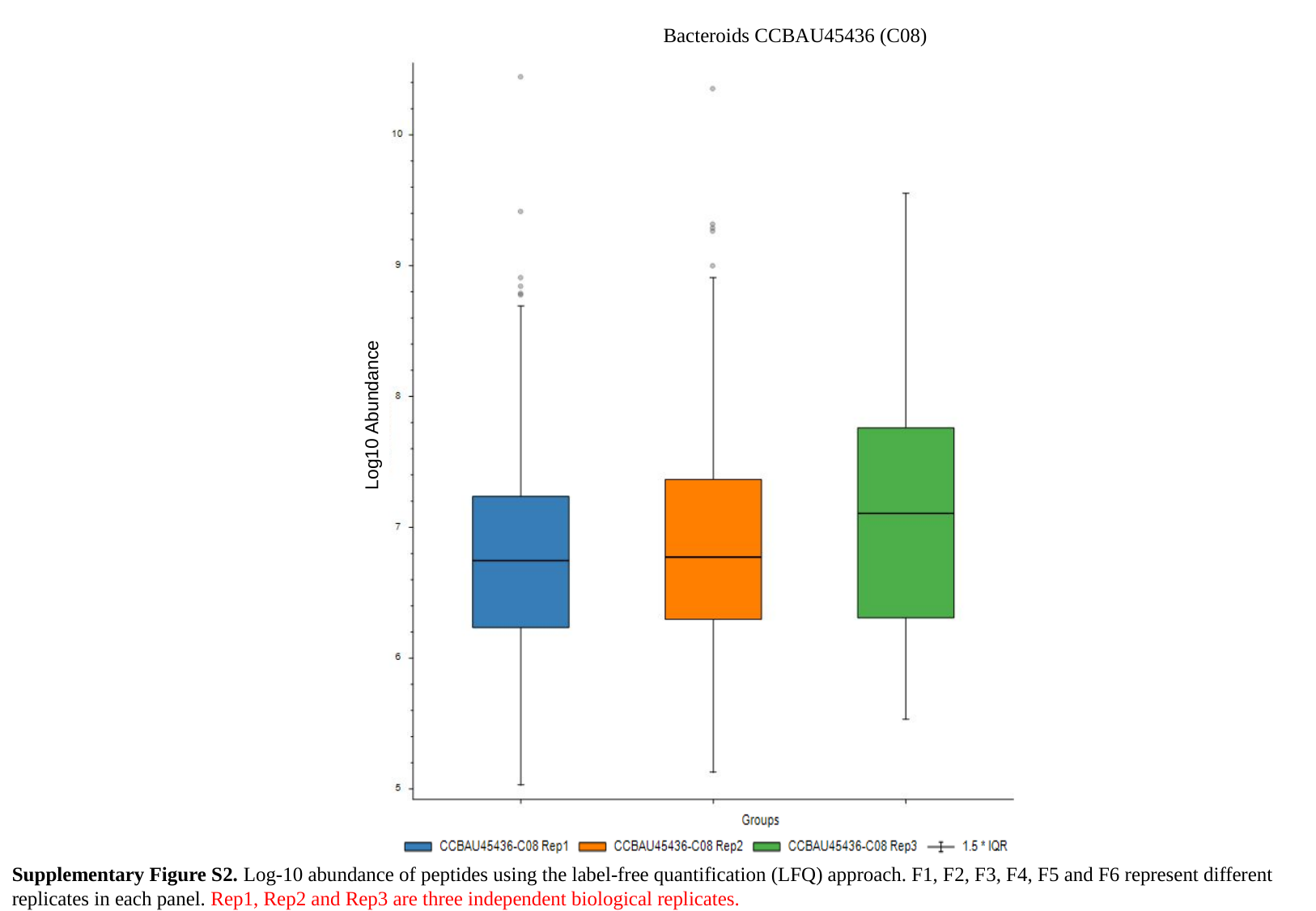

Bacteroids CCBAU45436 (C08)
Log10 Abundance
Supplementary Figure S2. Log-10 abundance of peptides using the label-free quantification (LFQ) approach. F1, F2, F3, F4, F5 and F6 represent different replicates in each panel. Rep1, Rep2 and Rep3 are three independent biological replicates.

## Slide 6
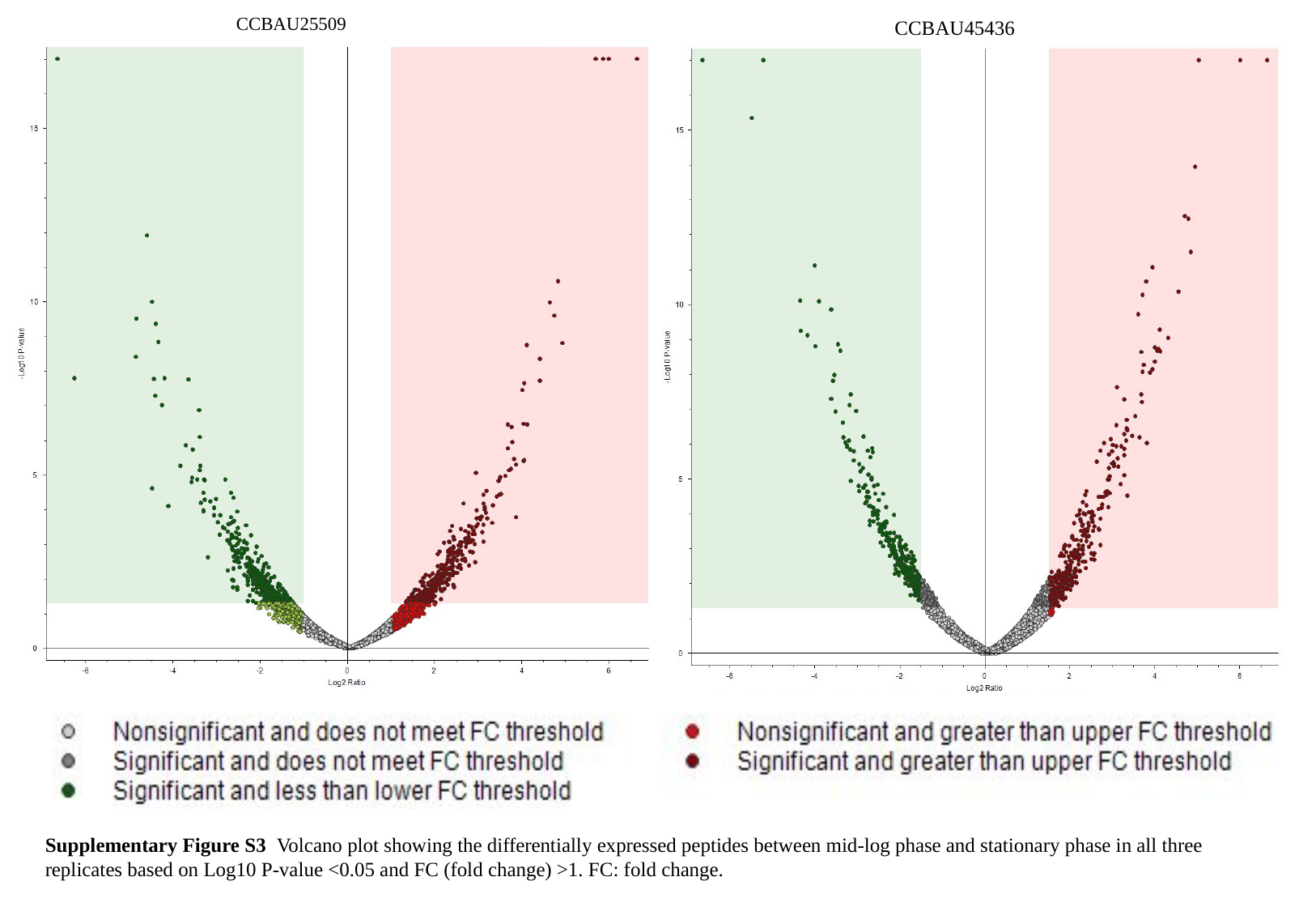

CCBAU25509
CCBAU45436
Supplementary Figure S3 Volcano plot showing the differentially expressed peptides between mid-log phase and stationary phase in all three replicates based on Log10 P-value <0.05 and FC (fold change) >1. FC: fold change.

## Slide 7
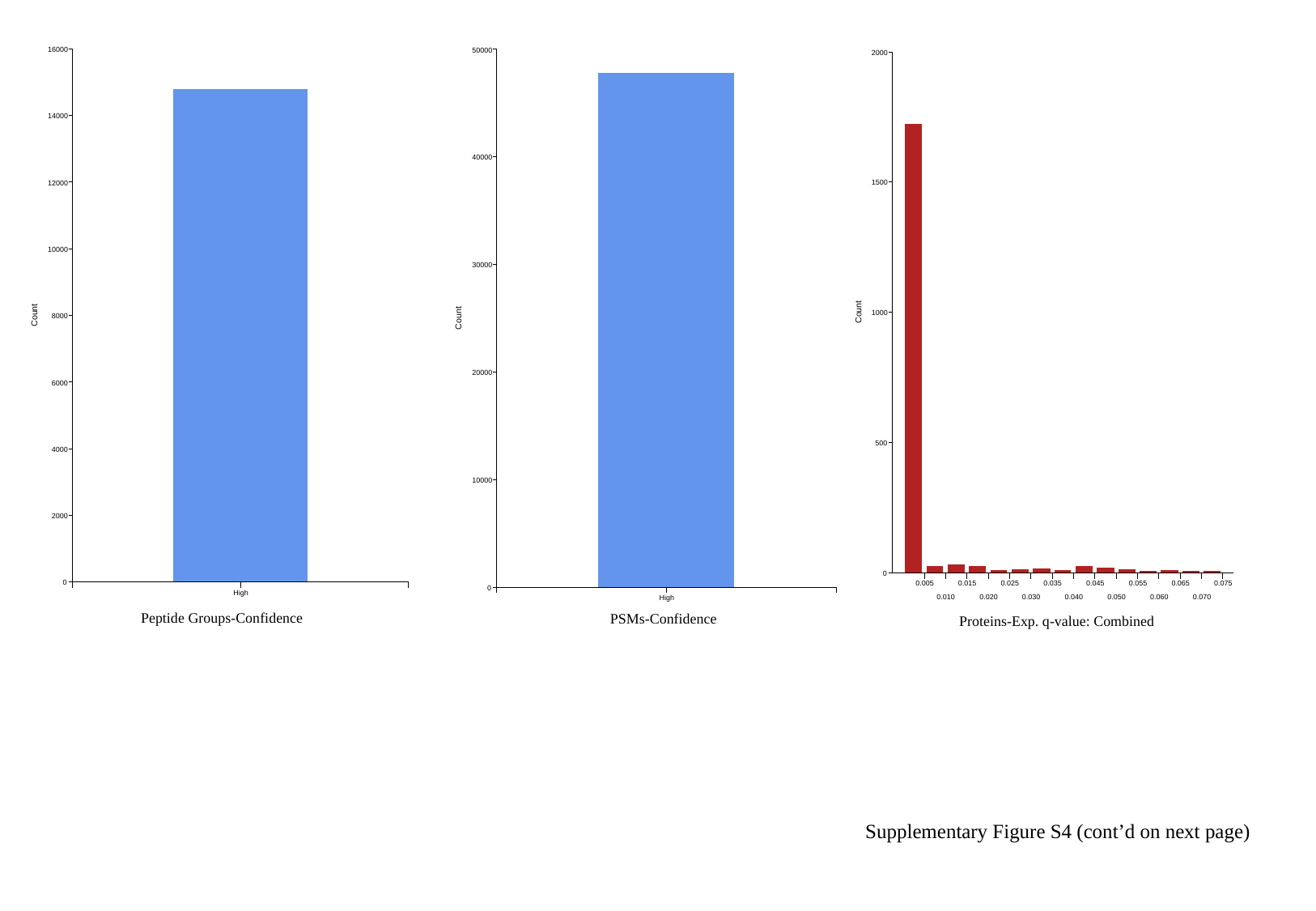

Peptide Groups-Confidence
PSMs-Confidence
Proteins-Exp. q-value: Combined
Supplementary Figure S4 (cont’d on next page)

## Slide 8
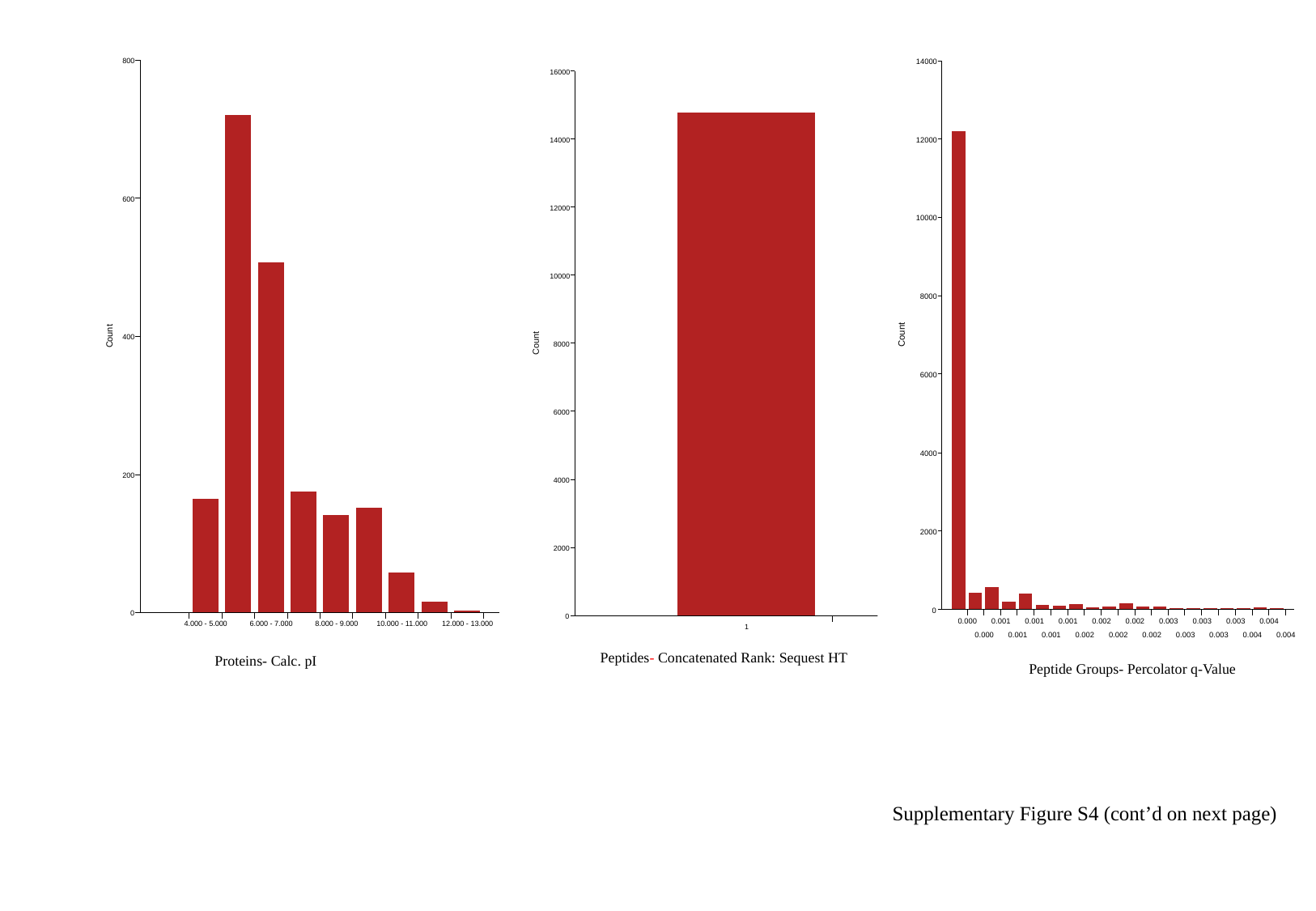

Peptides- Concatenated Rank: Sequest HT
Proteins- Calc. pI
Peptide Groups- Percolator q-Value
Supplementary Figure S4 (cont’d on next page)

## Slide 9
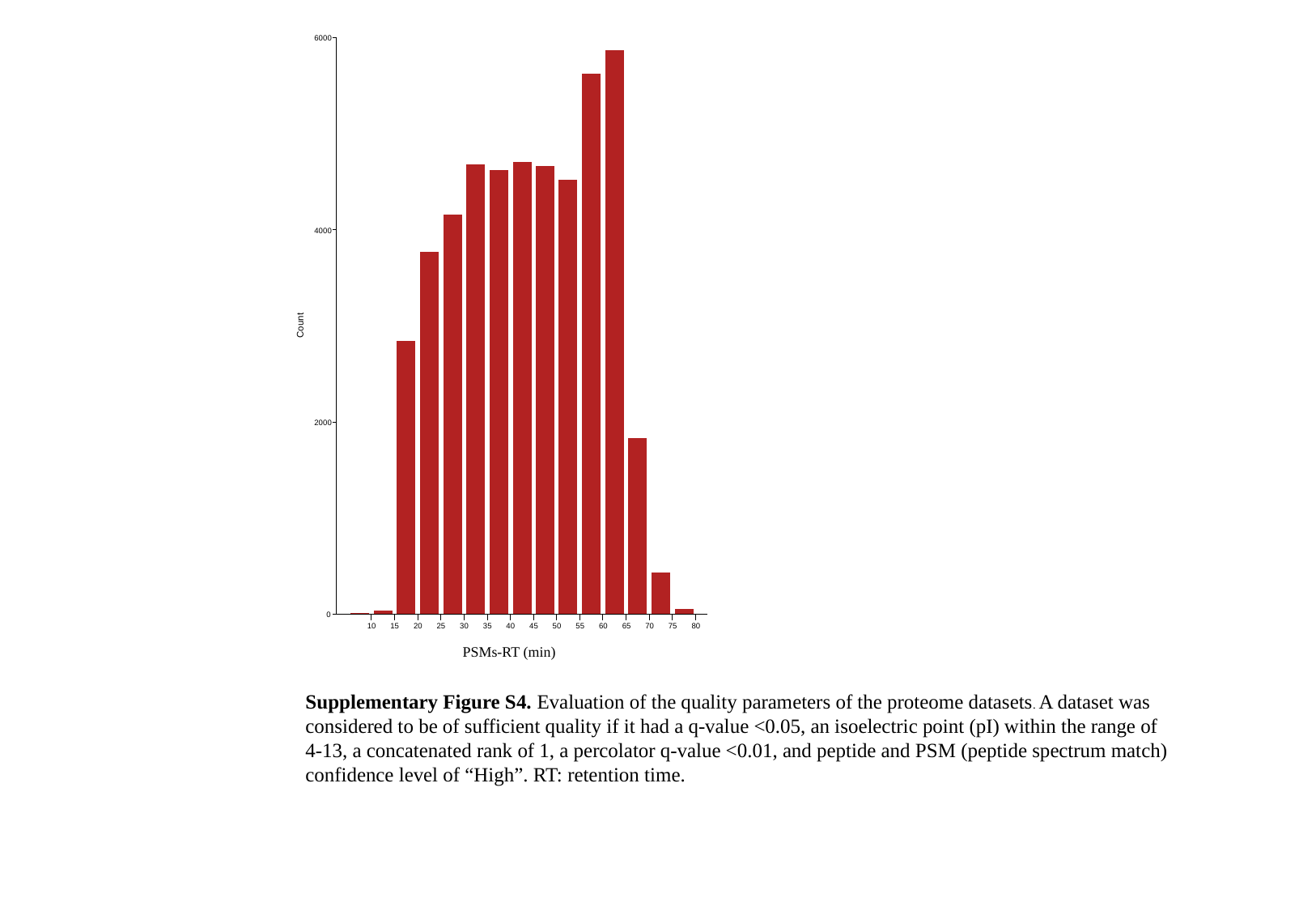

PSMs-RT (min)
Supplementary Figure S4. Evaluation of the quality parameters of the proteome datasets. A dataset was considered to be of sufficient quality if it had a q-value <0.05, an isoelectric point (pI) within the range of 4-13, a concatenated rank of 1, a percolator q-value <0.01, and peptide and PSM (peptide spectrum match) confidence level of “High”. RT: retention time.

## Slide 10
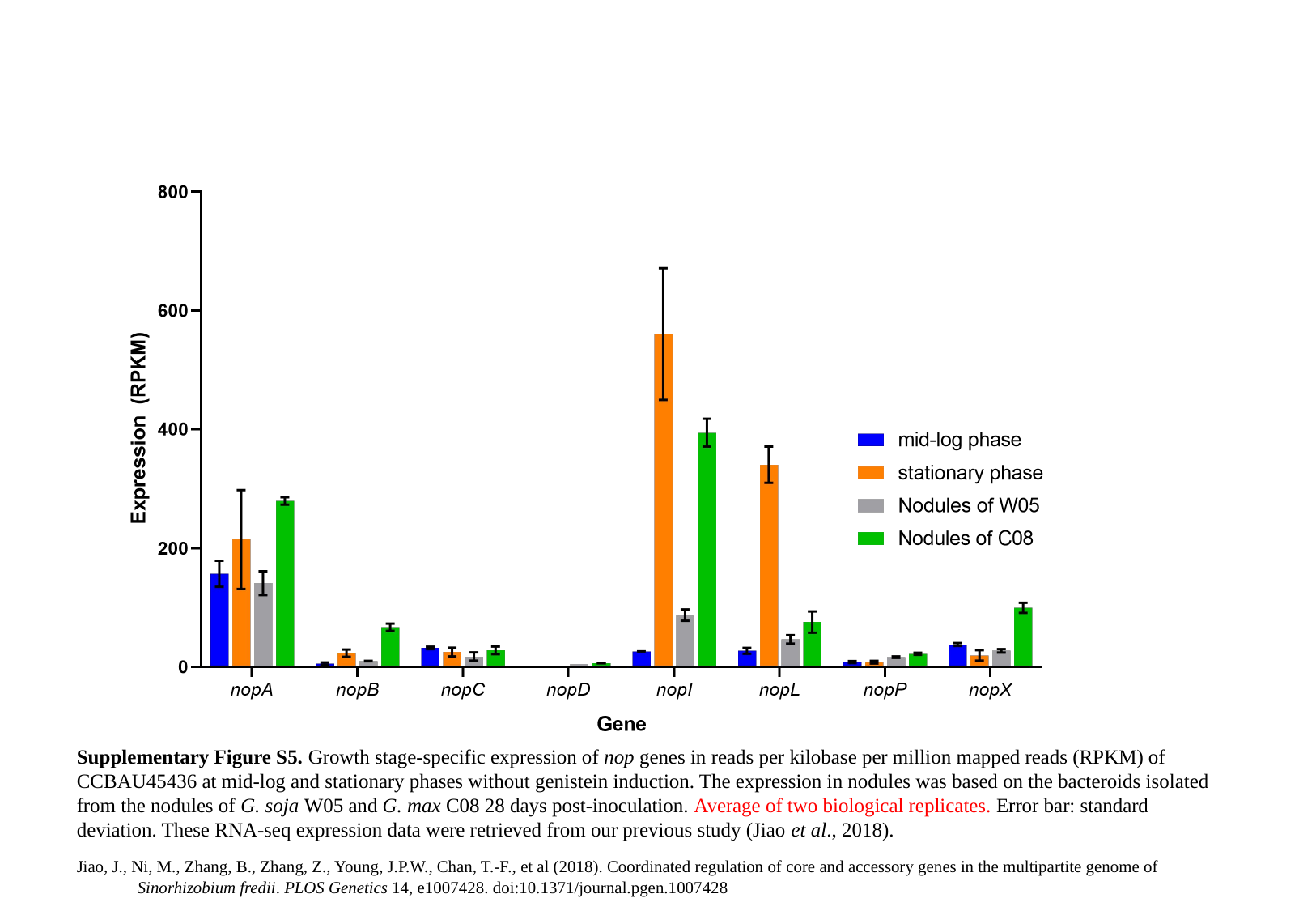

Supplementary Figure S5. Growth stage-specific expression of nop genes in reads per kilobase per million mapped reads (RPKM) of CCBAU45436 at mid-log and stationary phases without genistein induction. The expression in nodules was based on the bacteroids isolated from the nodules of G. soja W05 and G. max C08 28 days post-inoculation. Average of two biological replicates. Error bar: standard deviation. These RNA-seq expression data were retrieved from our previous study (Jiao et al., 2018).
Jiao, J., Ni, M., Zhang, B., Zhang, Z., Young, J.P.W., Chan, T.-F., et al (2018). Coordinated regulation of core and accessory genes in the multipartite genome of Sinorhizobium fredii. PLOS Genetics 14, e1007428. doi:10.1371/journal.pgen.1007428

## Slide 11
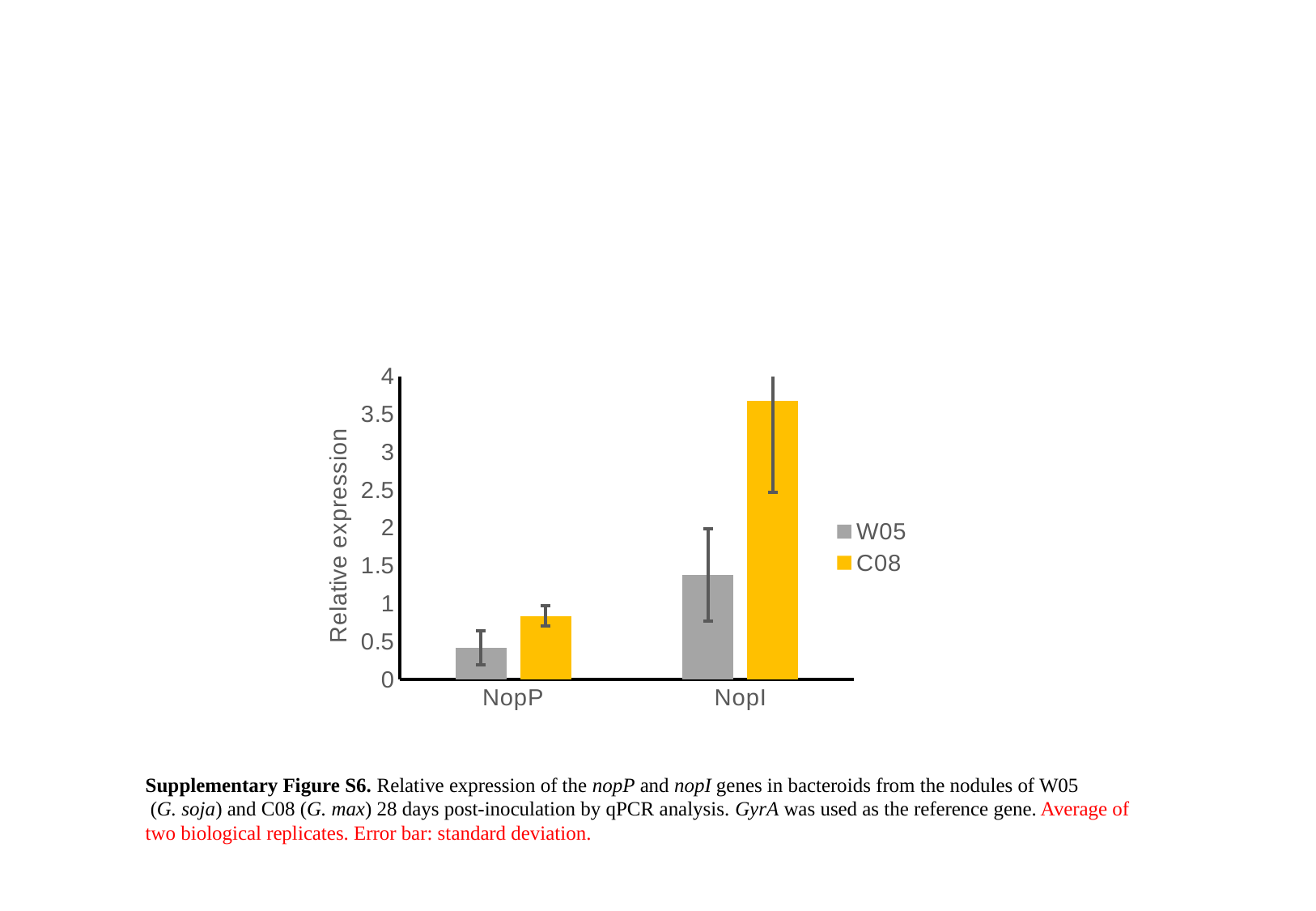

### Chart
| Category | | |
|---|---|---|
| NopP | 0.41612911986264994 | 0.8367963138593857 |
| NopI | 1.3780460996278527 | 3.6783091723379977 |Supplementary Figure S6. Relative expression of the nopP and nopI genes in bacteroids from the nodules of W05
 (G. soja) and C08 (G. max) 28 days post-inoculation by qPCR analysis. GyrA was used as the reference gene. Average of two biological replicates. Error bar: standard deviation.

## Slide 12
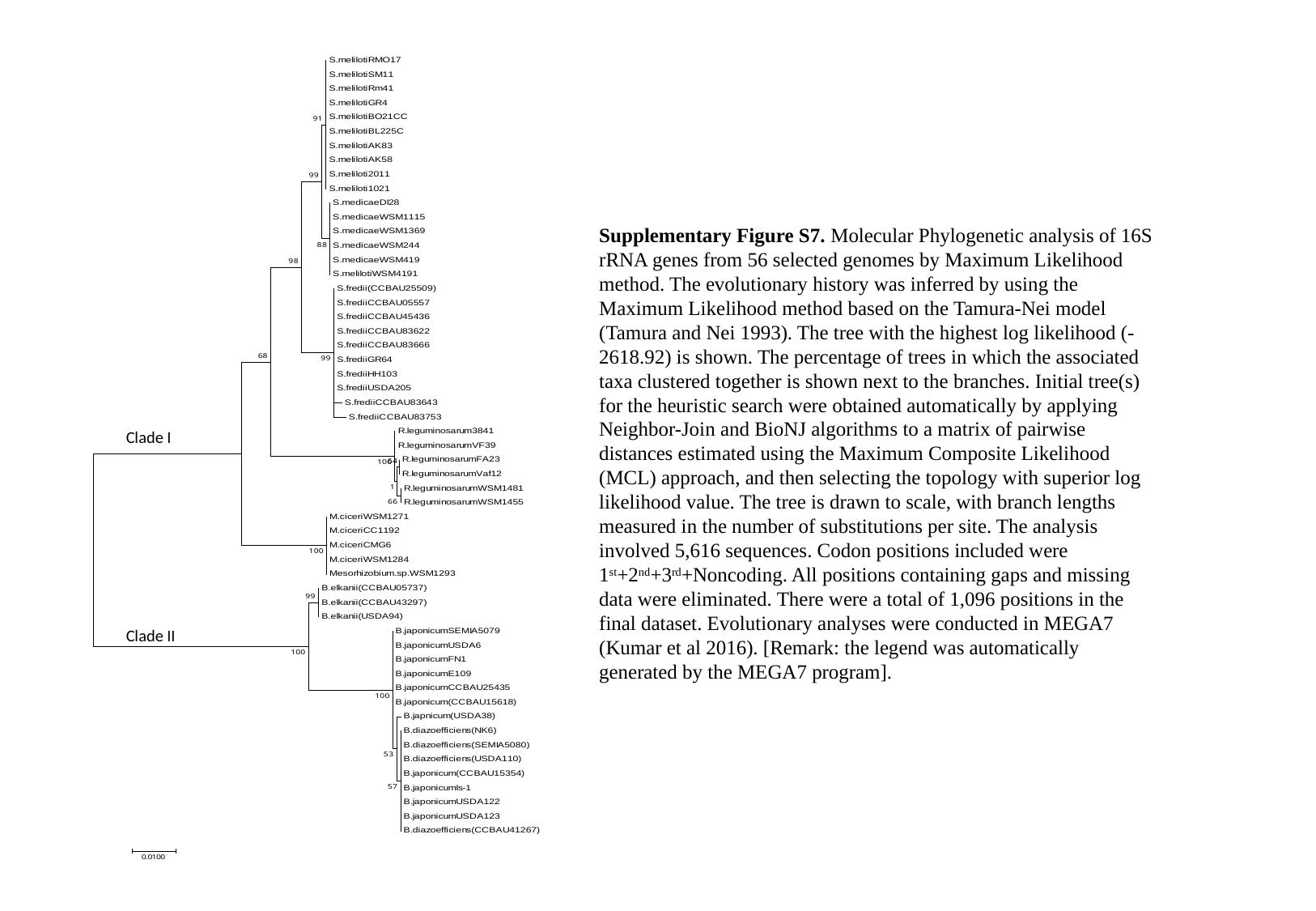

Clade I
Clade II
Supplementary Figure S7. Molecular Phylogenetic analysis of 16S rRNA genes from 56 selected genomes by Maximum Likelihood method. The evolutionary history was inferred by using the Maximum Likelihood method based on the Tamura-Nei model (Tamura and Nei 1993). The tree with the highest log likelihood (-2618.92) is shown. The percentage of trees in which the associated taxa clustered together is shown next to the branches. Initial tree(s) for the heuristic search were obtained automatically by applying Neighbor-Join and BioNJ algorithms to a matrix of pairwise distances estimated using the Maximum Composite Likelihood (MCL) approach, and then selecting the topology with superior log likelihood value. The tree is drawn to scale, with branch lengths measured in the number of substitutions per site. The analysis involved 5,616 sequences. Codon positions included were 1st+2nd+3rd+Noncoding. All positions containing gaps and missing data were eliminated. There were a total of 1,096 positions in the final dataset. Evolutionary analyses were conducted in MEGA7 (Kumar et al 2016). [Remark: the legend was automatically generated by the MEGA7 program].

## Slide 13
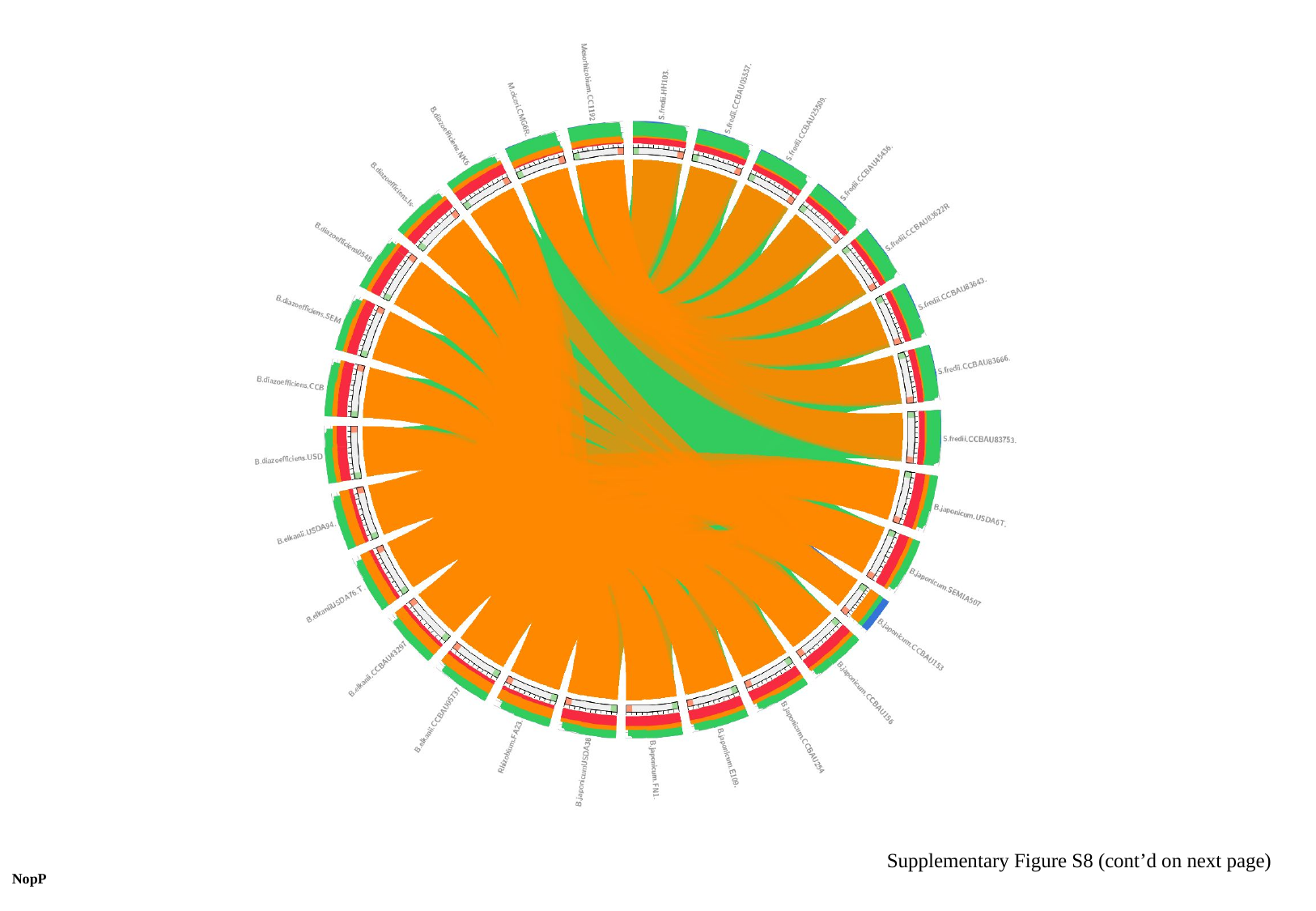

Supplementary Figure S8 (cont’d on next page)
NopP

## Slide 14
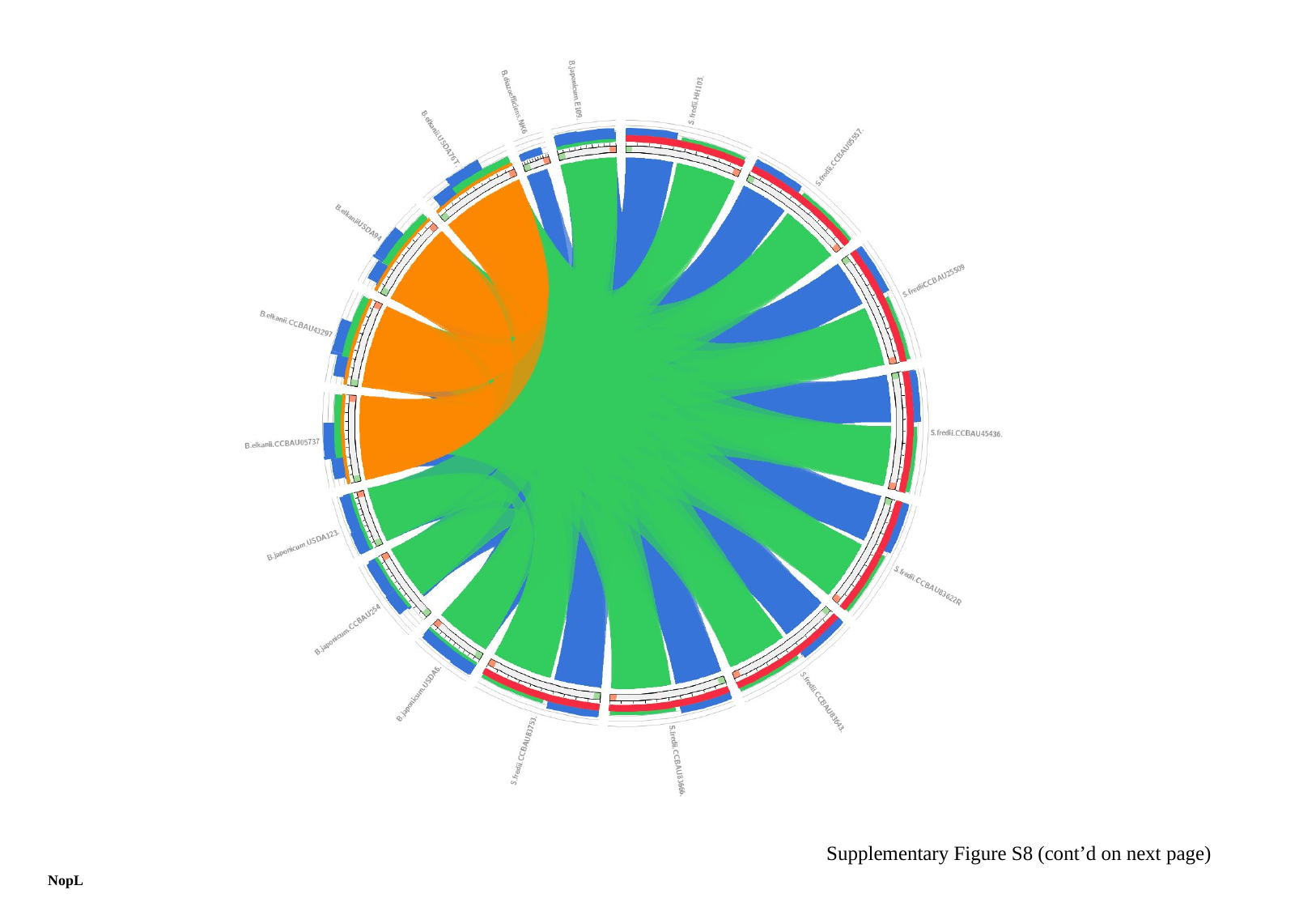

Supplementary Figure S8 (cont’d on next page)
NopL

## Slide 15
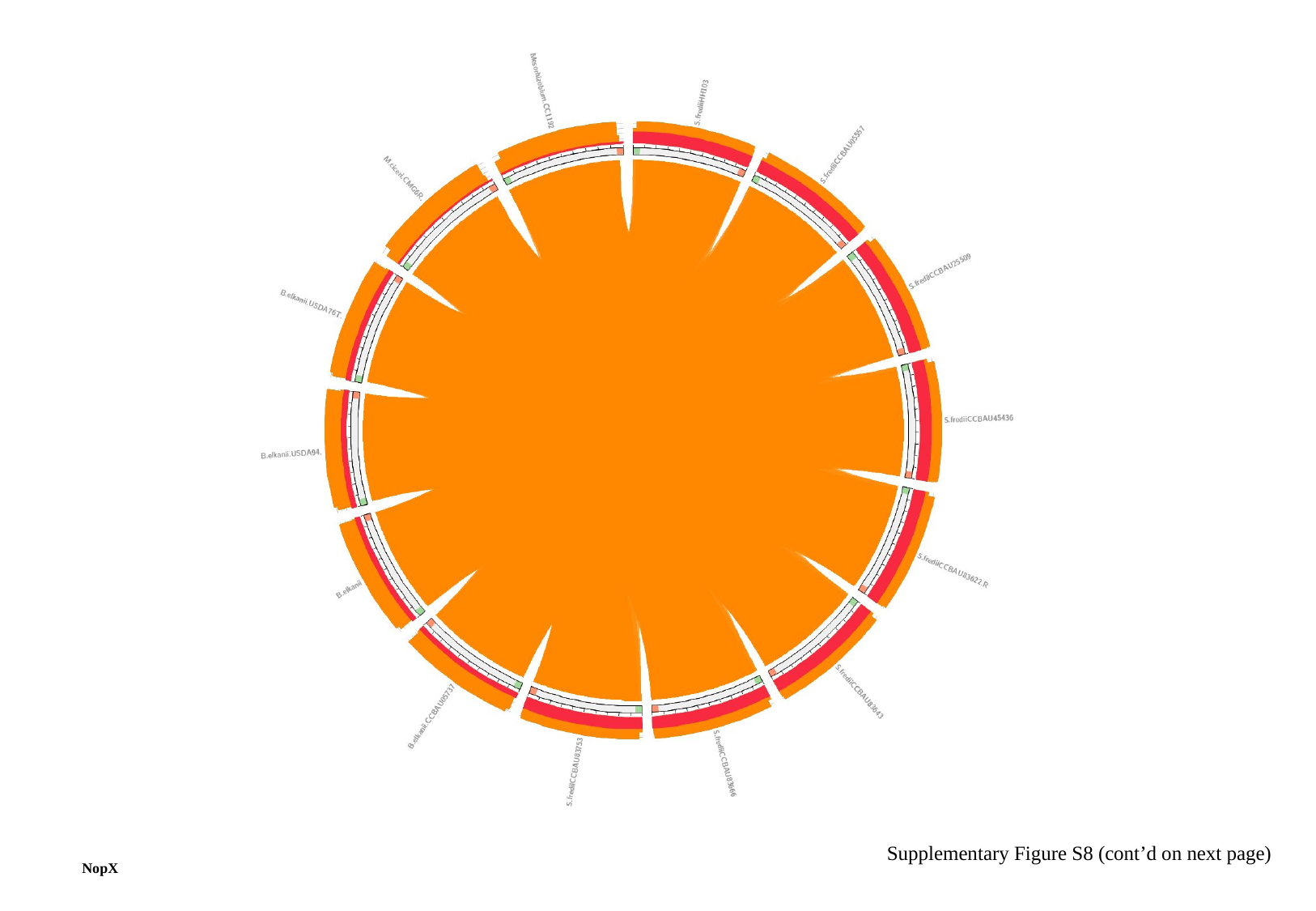

Supplementary Figure S8 (cont’d on next page)
NopX

## Slide 16
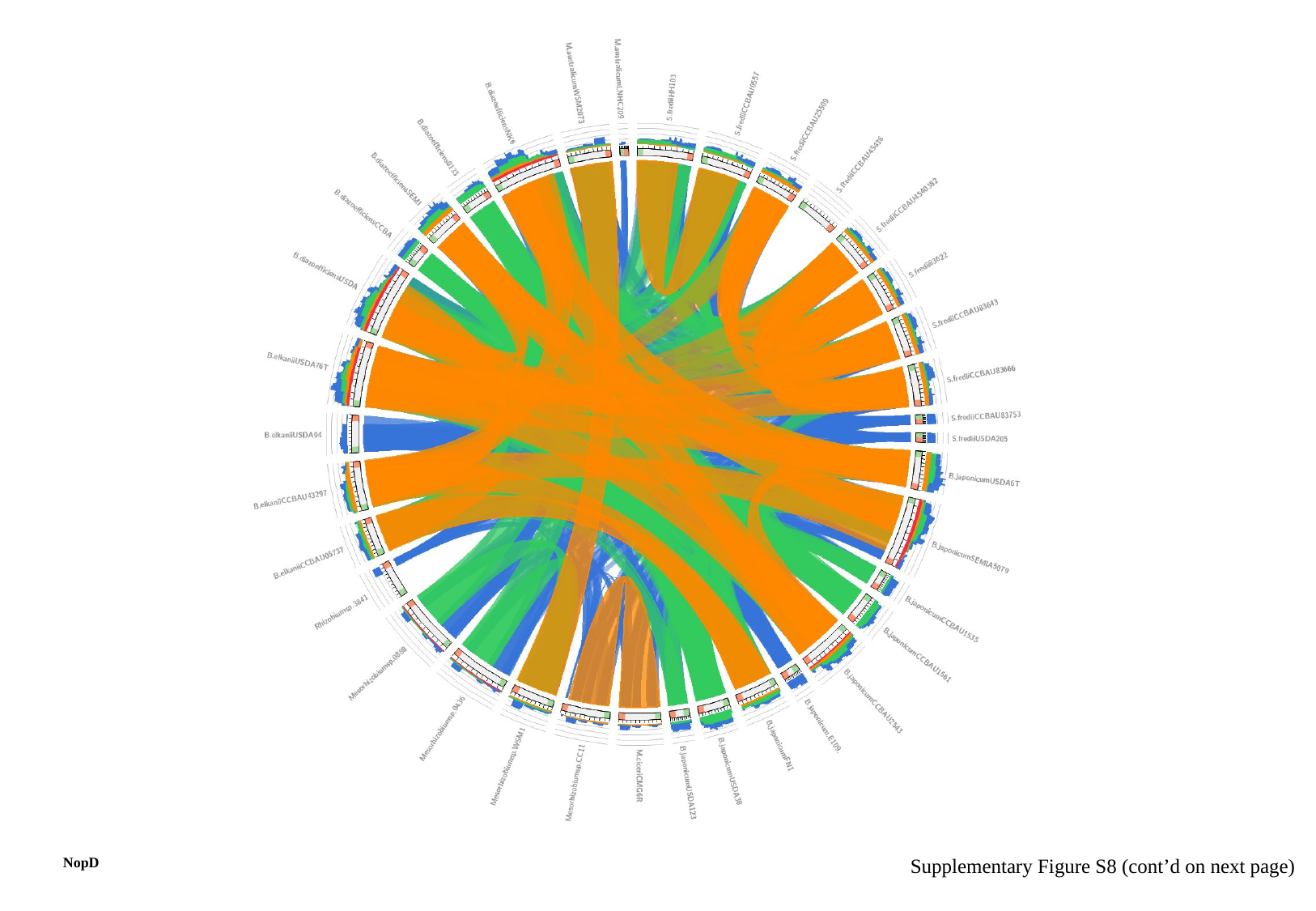

NopD
Supplementary Figure S8 (cont’d on next page)

## Slide 17
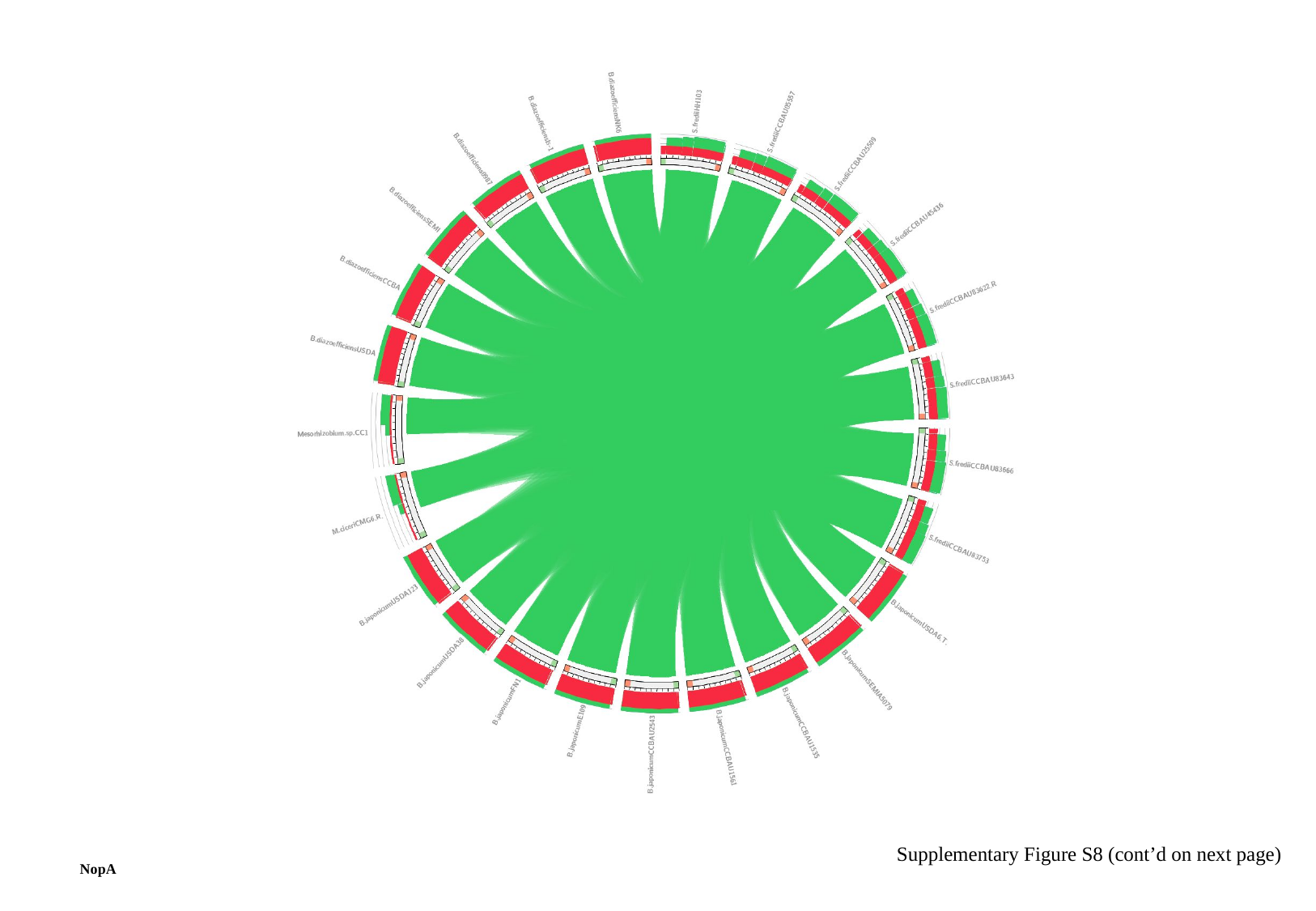

Supplementary Figure S8 (cont’d on next page)
NopA

## Slide 18
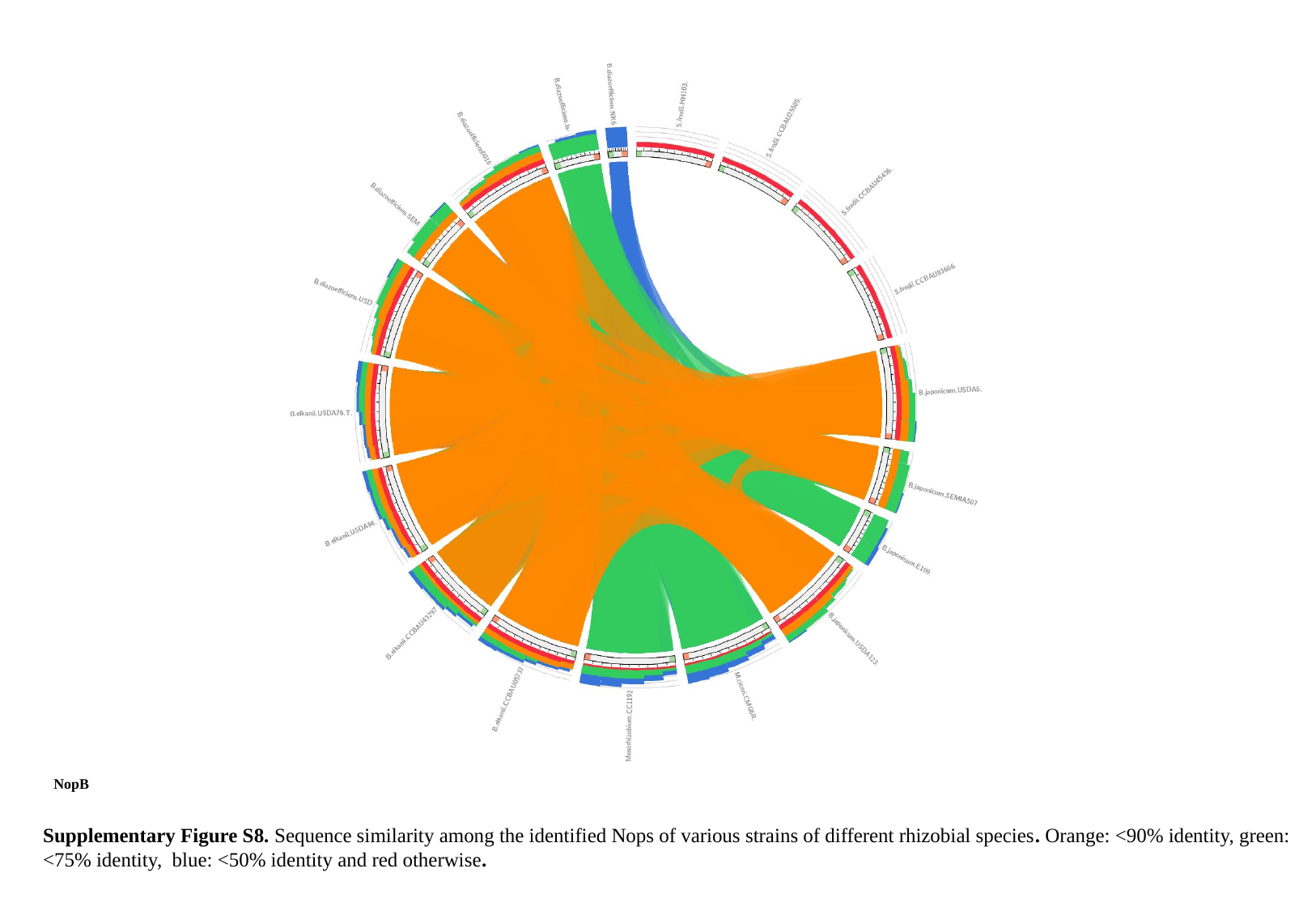

NopB
Supplementary Figure S8. Sequence similarity among the identified Nops of various strains of different rhizobial species. Orange: <90% identity, green: <75% identity, blue: <50% identity and red otherwise.

## Slide 19
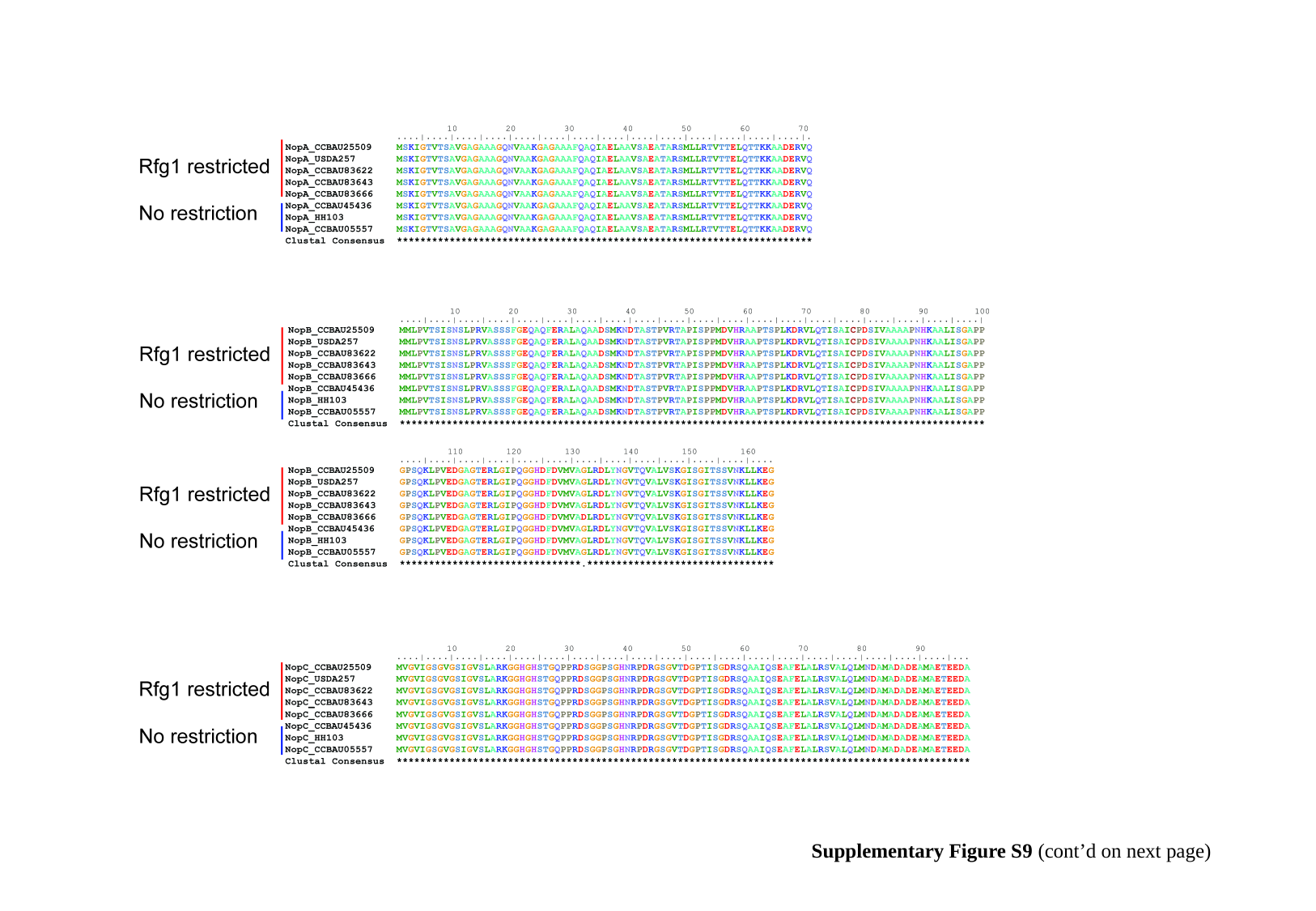

Supplementary Figure S9 (cont’d on next page)

## Slide 20
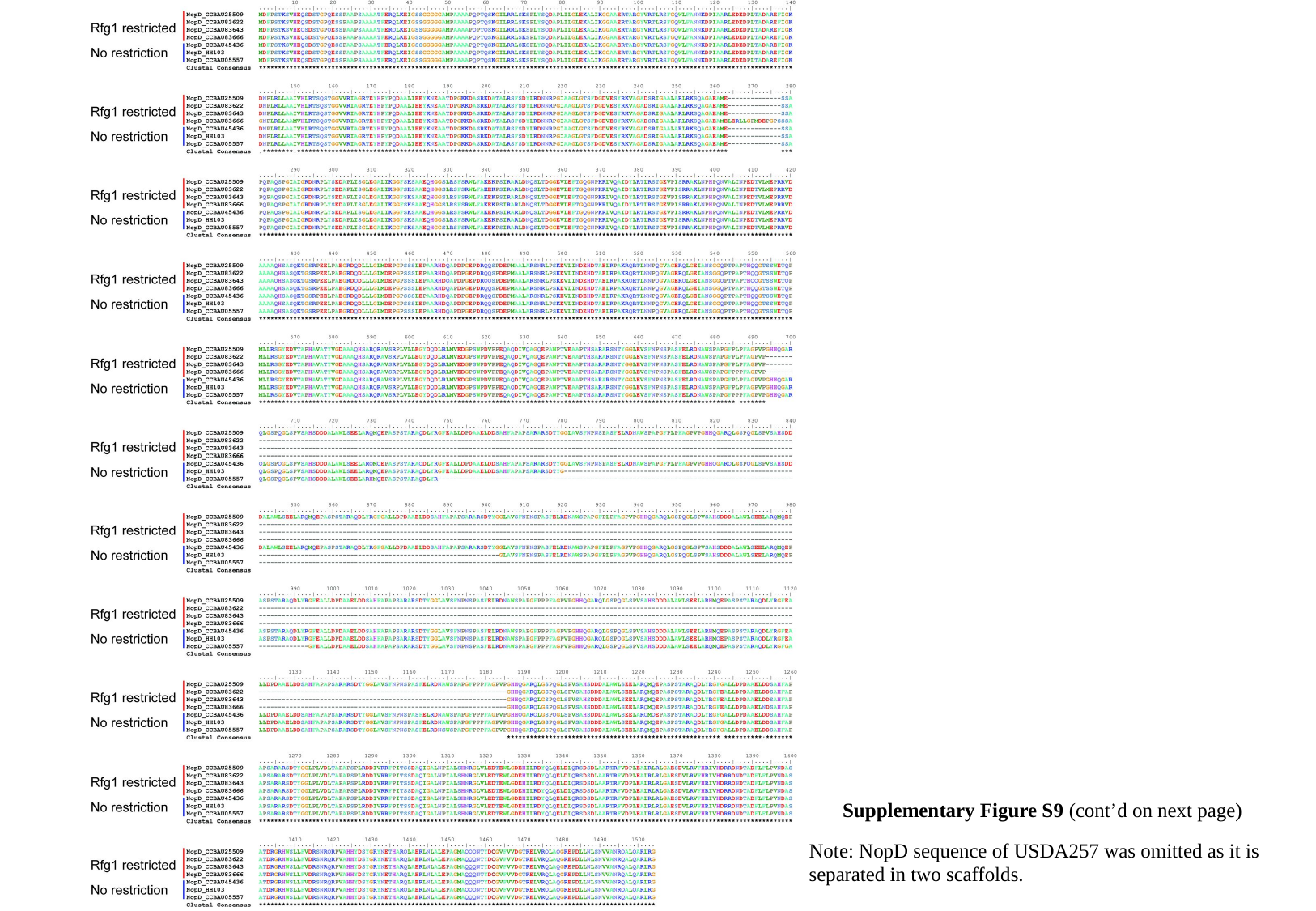

Supplementary Figure S9 (cont’d on next page)
Note: NopD sequence of USDA257 was omitted as it is separated in two scaffolds.

## Slide 21
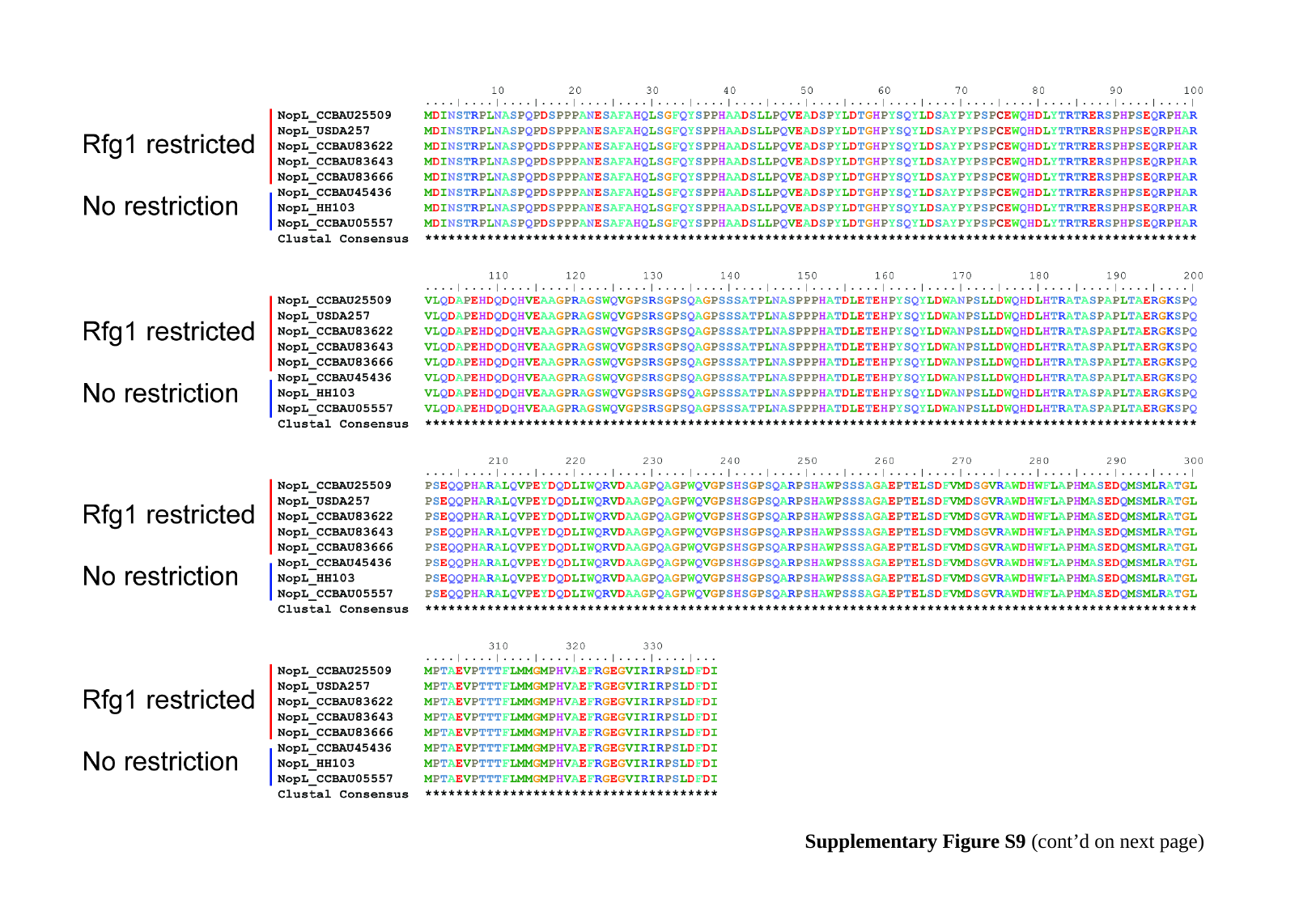

Supplementary Figure S9 (cont’d on next page)

## Slide 22
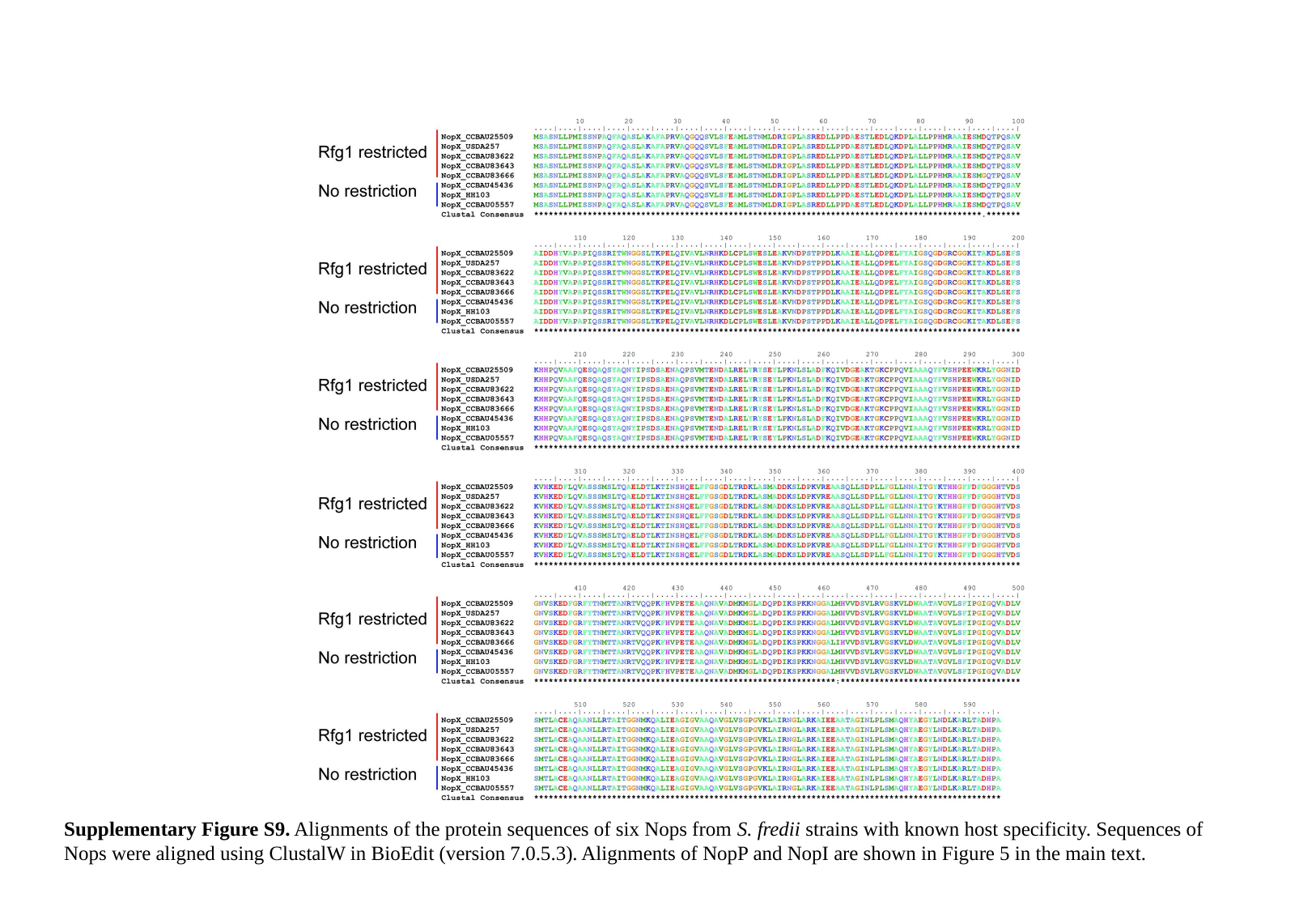

Supplementary Figure S9. Alignments of the protein sequences of six Nops from S. fredii strains with known host specificity. Sequences of Nops were aligned using ClustalW in BioEdit (version 7.0.5.3). Alignments of NopP and NopI are shown in Figure 5 in the main text.

## Slide 23
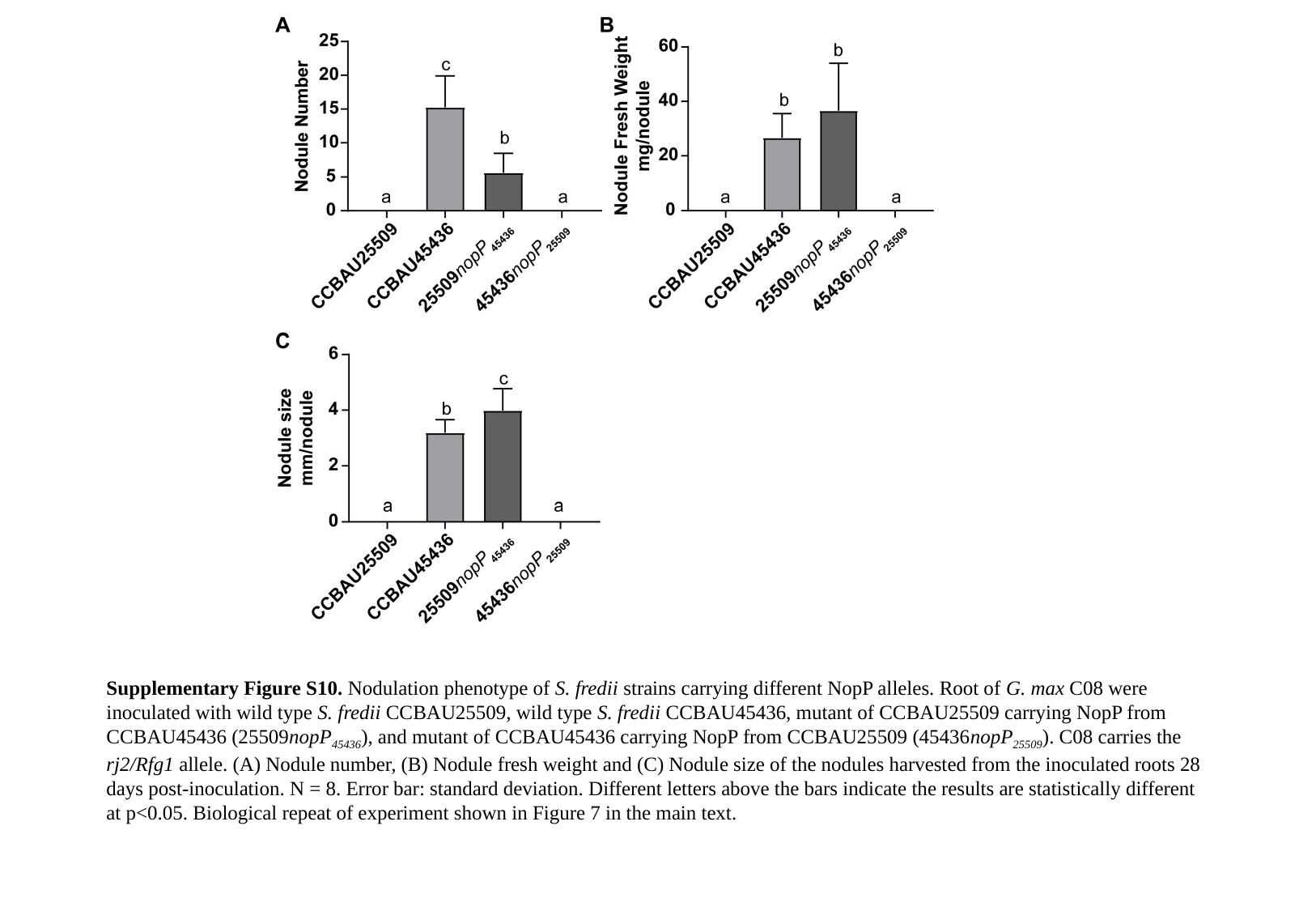

Supplementary Figure S10. Nodulation phenotype of S. fredii strains carrying different NopP alleles. Root of G. max C08 were inoculated with wild type S. fredii CCBAU25509, wild type S. fredii CCBAU45436, mutant of CCBAU25509 carrying NopP from CCBAU45436 (25509nopP45436), and mutant of CCBAU45436 carrying NopP from CCBAU25509 (45436nopP25509). C08 carries the rj2/Rfg1 allele. (A) Nodule number, (B) Nodule fresh weight and (C) Nodule size of the nodules harvested from the inoculated roots 28 days post-inoculation. N = 8. Error bar: standard deviation. Different letters above the bars indicate the results are statistically different at p<0.05. Biological repeat of experiment shown in Figure 7 in the main text.

## Slide 24
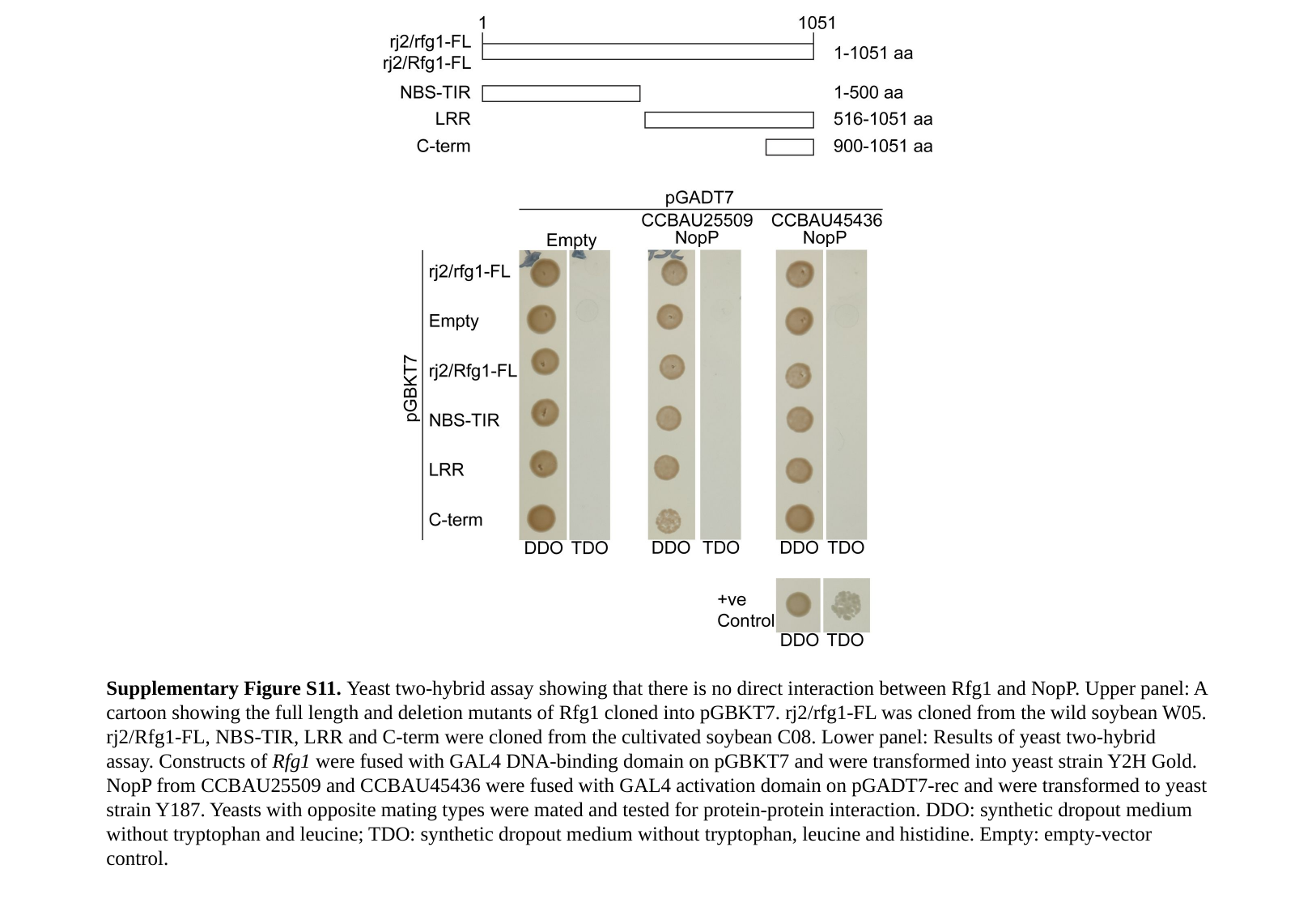

Supplementary Figure S11. Yeast two-hybrid assay showing that there is no direct interaction between Rfg1 and NopP. Upper panel: A cartoon showing the full length and deletion mutants of Rfg1 cloned into pGBKT7. rj2/rfg1-FL was cloned from the wild soybean W05. rj2/Rfg1-FL, NBS-TIR, LRR and C-term were cloned from the cultivated soybean C08. Lower panel: Results of yeast two-hybrid assay. Constructs of Rfg1 were fused with GAL4 DNA-binding domain on pGBKT7 and were transformed into yeast strain Y2H Gold. NopP from CCBAU25509 and CCBAU45436 were fused with GAL4 activation domain on pGADT7-rec and were transformed to yeast strain Y187. Yeasts with opposite mating types were mated and tested for protein-protein interaction. DDO: synthetic dropout medium without tryptophan and leucine; TDO: synthetic dropout medium without tryptophan, leucine and histidine. Empty: empty-vector control.
